# Projected Changes to Growth and Mortality of Hawaiian Corals over the Next 100 Years

**DOI:** 10.1371/journal.pone.0018038

**Published:** 2011-03-29

**Authors:** Ron K. Hoeke, Paul L. Jokiel, Robert W. Buddemeier, Russell E. Brainard

**Affiliations:** 1 Joint Institute for Marine and Atmospheric Research (JIMAR), University of Hawaii, Honolulu, Hawaii, United States of America; 2 Coral Reef Ecosystem Division (CRED), NOAA Pacific Islands Fisheries Science Center, Honolulu, Hawaii, United States of America; 3 Hawaii Institute of Marine Biology, University of Hawaii, Kaneohe, Hawaii, United States of America; 4 Kansas Geological Survey, Lawrence, Kansas, United States of America; Universidade de Vigo, Spain

## Abstract

**Background:**

Recent reviews suggest that the warming and acidification of ocean surface waters predicated by most accepted climate projections will lead to mass mortality and declining calcification rates of reef-building corals. This study investigates the use of modeling techniques to quantitatively examine rates of coral cover change due to these effects.

**Methodology/Principal Findings:**

Broad-scale probabilities of change in shallow-water scleractinian coral cover in the Hawaiian Archipelago for years 2000–2099 A.D. were calculated assuming a single middle-of-the-road greenhouse gas emissions scenario. These projections were based on ensemble calculations of a growth and mortality model that used sea surface temperature (SST), atmospheric carbon dioxide (CO_2_), observed coral growth (calcification) rates, and observed mortality linked to mass coral bleaching episodes as inputs. SST and CO_2_ predictions were derived from the World Climate Research Programme (WCRP) multi-model dataset, statistically downscaled with historical data.

**Conclusions/Significance:**

The model calculations illustrate a practical approach to systematic evaluation of climate change effects on corals, and also show the effect of uncertainties in current climate predictions and in coral adaptation capabilities on estimated changes in coral cover. Despite these large uncertainties, this analysis quantitatively illustrates that a large decline in coral cover is highly likely in the 21^st^ Century, but that there are significant spatial and temporal variances in outcomes, even under a single climate change scenario.

## Introduction

Anthropogenic climate change has created a dual global threat to reef-building scleractinian corals: (1) mass mortality due to increasingly frequent high temperature events (coral bleaching) and (2) decreased calcification rates due to increasing atmospheric carbon dioxide (CO_2_^atm^) that causes decreasing aragonite saturation state (Ω_a_) in surface waters (i.e. ocean acidification) [1], [2]. Because of coral adaptation to long-term Late Holocene environmental conditions, and also because of local variations in community composition and site-specific environments, regional modeling is the most practical way to bridge the scale mismatch between global climate projections and local reef responses.

Local managers, largely unable to affect global anthropogenic emissions policies, have little recourse but to attempt embrace strategies to sustain resilience of coral reef ecosystems so as to reduce impacts and slow ecological shifts to different (non-coral dominated) conditions [1], [2], [3], [4]. Knowledge of the magnitude and timing of these dual threats, which are likely to vary between locations, is necessary to make informed management decisions. There have been many quantitative estimates of projected climate change driving increasing temperature-related (coral bleaching) episodic mortality and modeling associated susceptibility [5], [6], [7], [8], but few attempts to model the role of ocean acidification and increasing temperature on coral growth; including the calculation of recovery potential from episodic mortality events (e.g. [9], [10]).

In this analysis, we attempt to evaluate the dual threats to corals by extending the Coral Mortality and Bleaching Output (COMBO) model [9]. Similar to the COMBO model, the extended model utilizes predicted sea temperature, predicted CO_2_^atm^, observed coral growth (calcification) rates, and observed mortality linked to mass coral bleaching episodes. However it diverges most from previous studies by providing multiple predictions of future conditions: multiple runs of 20 structurally-different Atmosphere-Ocean General Circulation Models (AOGCMs) and a separate Monte Carlo approach are used to provide separate predictions of sea surface temperature (SST) and Ω_a_. This provides multiple realizations and establishes multi-model (ensemble) means with a range of possible outcomes (a measure of uncertainty) specific to each study location. In other climate studies, this multi-model approach has shown better large-scale agreement with observations, because individual model biases tend to cancel. Ensembles of projections of future change therefore provide higher quality and more quantitative change information [11]. 

This pilot study focuses on sites within the greater Hawaiian Archipelago to allow an examination of model sensitivities in a region of relatively low biological diversity (compared with the western Indo-Pacific) and reasonably well-studied responses of growth rates of several dominant reef-building corals to temperature [9], [12], [13], [14]. These combine to reduce complexities caused by inter-genus differences in coral metabolism and varying responses to temperature changes, while still covering a significant climate gradient (over 10° of latitude 25° of longitude). The study is also focused on the IPCC AR4 future emission scenario A1B [15] because it is roughly in the middle of the range of the AR4 future emission scenarios, and is the scenario for which the greatest number of AOGCM realizations is available. The authors make no assumption that this is the most likely future scenario. The simplifying restrictions of location and future emissions scenarios reduce confounding variables, better allowing evaluation and sensitivity testing of the model and better examination of the overall combined effects of ocean warming and acidification.

**Figure 1:**
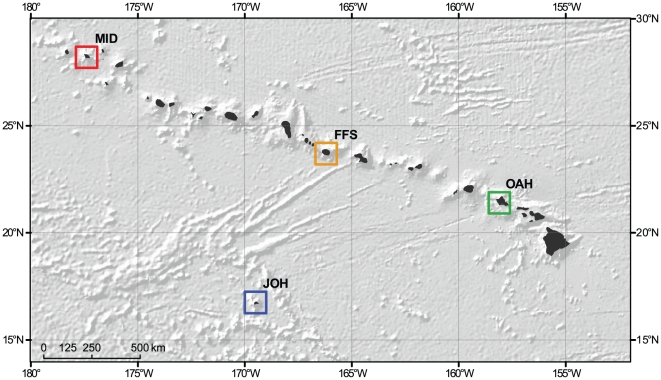
Greater Hawaiian Archipelago. Colored boxes represent 1°×1° boxes around Johnston Atoll (JOH), the Island of Oahu (OAH), French Frigate Shoals (FFS), and Midway Atoll (MID); these coincide with historical SST data and the reference location for AOGCM data extraction for each location.

The projections of coral cover change at Midway Atoll (MID), French Frigate Shoals (FFS), Oahu (OAH) and Johnston Atoll (JOH) presented here serve as proxies for their respective general areas of the Hawaiian Archipelago (Fig. 1). While JOH is arguably not geologically part of the archipelago, it has been included due to its well-documented biological connectivity to it [16] and to provide a broader geographic range.

The modeling techniques presented here account for predicted changes in SST and Ω_a_ at the spatial scale of the data used to downscale the AOGCMs (on the order of one degree of latitude). Other factors with potentially large effects, such as local anthropogenic impacts, changes in oceanographic conditions (such as ocean stratification and storm activity), and local reef morphology are either not included in or are poorly represented in the AOGCMs. Furthermore, the model does not account for various aspects of ecosystem dynamics, such as taxonomic succession or local carbonate chemistry processes. To attempt to model these factors at the current state of understanding of coral ecosystem response to anthropogenic impacts would likely confound any attempts to elucidate the direct impacts of increasing water temperatures and decreasing Ω_a_ alone. As such, the projections presented here should not be considered quantitative forecasts of percent coral cover change at specific locations; rather, they should be viewed as broad-scale probability-based estimates of the relative impact of predicted increases in SST and CO_2_ to overall coral growth in different regions of the Hawaiian Archipelago over the next ∼100 years (until 2100 A.D.). This model analysis shows how latitudinal differences may lead to large relative differences in coral growth/coral cover across an archipelago and highlights the need to better understand the ability of corals to adapt or acclimate to increasing frequency of episodic heat stress events and the associated levels of mortality if coral cover trajectories are to be estimated.

## Methods

The Coral Mortality and Bleaching Output (COMBO) model was extended by: (1) automating the use of multiple Atmosphere-Ocean General Circulation Models (AOGCMs) rather than a single Simple Climate Model (SCM) as input (for concise definitions, see [11]); and (2) by replacing the model's existing coral bleaching module with a more process-based module trained by observations of mortality associated with past bleaching events and based on both the seasonal variability expressed by the different AOGCMs and historical data at specific study locations.

The two methods of AOGCM input were: (1) based strictly upon the multiple AOGCM input, and (2) a Monte Carlo approach of seasonal variability around long term (decadal) temperature trends. These two methods are termed the ‘individual model ensemble’ and the other ‘model ensemble Monte Carlo simulation’ respectively, and both provide multiple change predictions derived from multiple AOGCMs as input, resulting in multiple realizations of possible future changes in coral cover using a variety of model parameters. The two resulting ensembles provide a range of possible outcomes and a central tendency for each location studied. The sections below outline (1) methods and assumptions used in the coral cover change model; (2) selection and preparation of SST and CO_2_^atm^; and (3) ensemble member generation for both methods. AOGCM input to both methods utilizes IPCC emission scenario A1B [15]. Additionally, model validation utilizes “climate of the 20^th^ century” emission scenario 20C3M [15].

### Coral cover change model

With the exception of the episodic mortality event module (see below), all module algorithms used were based on the COMBO model; for more details on equations and associated assumptions, refer to [9]. A brief overview of the modules and some of the assumptions used are given below:

#### Long term coral growth and mortality module

**Figure 2:**
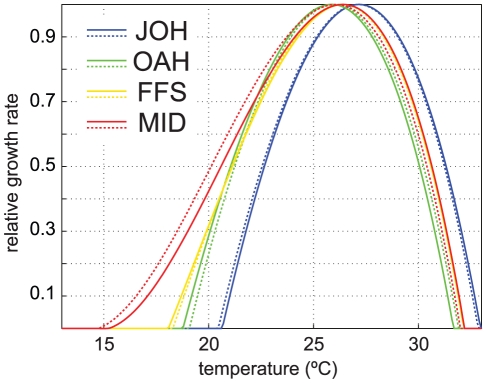
3rd-order polynomial used to calculate relative coral growth curves at study locations. Maximum growth occurs at maximum climatological mean monthly temperature – 2 standard deviations; minimum growth at minimum/maximum mean monthly temperatures ±5°C. Solid lines represent climatological values derived from ERSST v3; dotted lines from Pathfinder SST v5.

This module estimates coral growth rates based on temperature. Annual long-term coral growth and mortality rates were assumed to be at equilibrium at the start of the model run (year 2000), i.e. the increase in coral cover due to recruitment and growth equals losses due to mortality and no net change in coral cover is occurring. Relative coral growth rates were calculated by solving a 3^rd^-order polynomial in which maximum net growth (G_max_) is assumed to occur when:
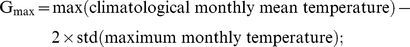
where “max” and “std” are the maximum and standard deviation of the enclosed quantities, respectively. Zero net growth (G_min_) is assumed to occur when:
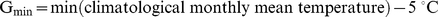
and
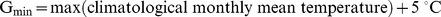
This equation results from a best-fit of values from laboratory and field observations of coral growth for Hawai'ian reef corals *Pocillopora damicornis*, *Montipora capitata*, and *Porites lobata* [12], [13], [14]), and tested to see if scaling to other temperature regimes and species lead to an acceptable fit (e.g. [17], [18]). For the development and site-specific application of the model growth curves, please refer to [9]. Relative growth curves used here are plotted for the four study locations in Fig. 2.

#### Long-term CO_2_ effects module

This module estimates Ω_a_ and resulting changes in coral calcification rate. For model, it is assumed that pCO_2_ in the surface ocean equilibrates with CO_2_^atm^ on an approximately annual time scale [19]. The BERN2.5CC model [20] is used to estimate atmospheric CO_2_ concentration. Ω_a_ is estimated from temperature and pCO_2_ values through interpolation of the pCO_2_-temperature-Ω_a_ saturation values based on methods outlined by Kleypas et al. [21]. The sensitivity of corals to changes in Ω_a_ is defined as a decrease in growth per unit decrease in Ω_a_. This is an adjustable coefficient in the model; for example, a coefficient of 0.3 will result in a linear decrease in (coral) calcification rate of approximately 30% for a decrease in Ω_a_ from 3.8±0.2 in 1999 to 2.5±0.2 in 2099. This level of sensitivity is suggested by Kleypas et al. [21] and Langdon et al. [22], and represents a rough average of a number of other studies summarized by Kleypas and Lagndon [23].

#### Episodic heat stress mortality event module

**Figure 3:**
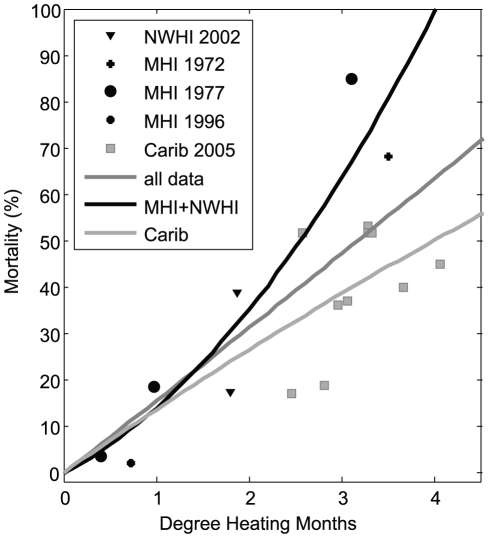
Degree Heating Months (DHM)/coral mortality relationships used to calculate mortality from episodic heat stress (coral bleaching) events. Observations of colony mortality associated with the 2005 event in the eastern Caribbean (‘Carib05’ as compiled by Buddemeier et al. in review); the 2002 Northwestern Hawaiian Islands event (‘NWHI02, Kenyon et al. 2006); observations of coral mortality associated with heated effluent (‘MHI72’, Jokiel and Coles 1974); a laboratory study of Hawaiian corals (‘MHI77’, Jokiel and Coles 1977), and a the 1996 main Hawaiian Islands event (‘MHI1996’, Jokiel and Brown 2004), are plotted for comparison. The curves ‘all data’, ‘MHI+NWHI’, and ‘Carib’ are 2^nd^ order best fits of all of the data points, only data associated events in the Hawaiian islands, and only data from the 2005 Caribbean event.

This module calculates eventual coral mortality associated with episodic bleaching events. Unlike the above modules, this module departs from the COMBO module. Rather than using a pre-set (user selected) number of events, the new method calculates annual degree heating months (DHM), a measure of heat exposure defined [5], [24] and others, directly from the temperature input itself. DHM here is defined as the sum of monthly temperature values above the maximum climatological monthly mean +1°C (sometimes known as the “bleaching threshold”); e.g. 2 months with an average temperature of 2°C above the maximum monthly mean results in 4 DHM. Mortality levels are then assigned to a particular level of DHM (estimated from a 2^nd^ order fit), based on mortality associated with mass bleaching events. Higher order fits between DHM and these data were tested, but either did not improve the goodness of fit, or lead to unrealistic results, such as a leveling of mortality rates at higher DHM. The mortality values attempt to include longer term mortality effects following a thermal stress event, such as increased susceptibility to disease (e.g. [30]), rather than immediate mortality directly due to the temperature stress itself. The ability to use a different starting and ending value for the “bleaching threshold” is included in the model; this attempts to model coral's ability to adapt to higher temperatures. Actual values for mortality rates were derived from observations of the Northwestern Hawaiian Islands 2002 and 2004 bleaching events [26], [27], the main Hawaiian Islands1996 bleaching event [28], [29], and laboratory studies of Hawaiian corals [14]. Although Caribbean reefs are dominated by different coral species than Hawaiian, observations of mortality rates associated with the 2005 Caribbean bleaching events (summarized by Buddemeier et al. [25]) were also included, as they are considered better documented than the mortality rates associated with Hawaiian events. These values and the associated best 2^nd^ order fits are plotted in Fig. 3.

### Input temperature transformation and historic dataset selection

Predicted SST was extracted from AOGCMs hosted by the World Climate Research Programme's (WCRP's) Coupled Model Intercomparison Project phase 3 (CMIP3) multi-model dataset [31]. Predicted SST is termed the temperature of surface, or TOS, in the CMIP3 database. For a full list of models and model runs selected from the database, see Table S1. Since the process-based AOGCMs may contain stochastic fluctuations (similar to the Earth's real climate), reconstructions of 20^th^ century climate may contain substantial biases compared with historic observations and differing sensitivities may result in unrealistic seasonal amplitudes [32]. Thus a significant discontinuity between predicted temperatures and historic temperatures frequently occurs, and predicted seasonal variability often departs from observations. It is therefore sometimes impossible to use AOGCM data to predict current and future coral growth rates and lethal coral bleaching events without first constraining their sensitivities to values closer to those observed during the 20^th^ century [5], [7], [8]. These transformations also serve to statistically downscale the climate predictions, a necessary step in examining processes at a scale finer than that of the climate prediction [33].

**Figure 4:**
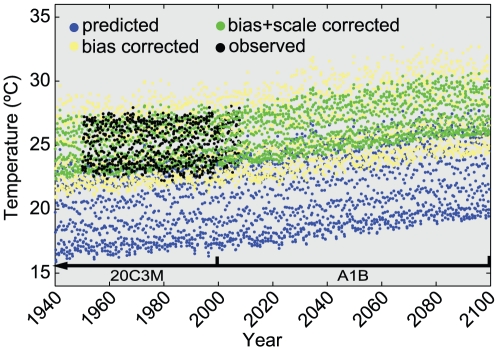
Example of bias correction and seasonal scaling of AOGCM data. The example in this case is FFS; temperature predictions for Scenarios 20C3M and A1B from the CSIRO-Mk3.5 model (blue points) are first bias corrected (yellow points), and then seasonally scaled (green points), with observed temperature data (ERSST v3, black points).

The transformation method used here maintain the prediction's native variation and net increase, but scales it so that mean seasonal fluctuations match those of the historical during the period of overlap. An example is shown in Fig. 4. First, the mean bias and mean difference between the seasonal amplitudes of the AOGCMs of the 20^th^ century (years 1900–1999, Scenario 20C3M) and the historical observations at each location were found. Seasonal scaling was then performed as follows: fourth-order polynomial fits were computed for both the model and historic time series at each location and then subtracted from their respective original time series, creating two sets of seasonal residuals. Normal cumulative distribution function probabilities were then calculated for model residuals, and then inverted, replacing sigma (standard deviation) values from the historic residuals. Higher order fits were tested, but did not improve characterization of seasonal variability (in the least-squares sense); non-normal probability distributions likewise did not lead to better characterization. The calculated biases and seasonal scales, specific to each location and each model in the multi-model database, were then applied to the same respective locations and model for future Scenario A1B (years 2000–2099). These methods used to downscale the AOGCM-predicted SST follows that of Sheppard [7] and Sheppard and Rioja-Nieto [8].

In this study, three historical temperature datasets were considered: Pathfinder SST ver.5 [34], ERSST ver.3 [35], and HadISST ver.1.1 [36] for use in the calculation of the following: growth curves (long-term coral growth and mortality module), DHM thresholds (episodic temperature event module), transformation of predicted SST, and probability density functions of seasonal (monthly) temperature variability. Although the satellite-based Pathfinder is in most aspects a superior data set in terms of consistency, precision and accuracy, ERSST was chosen as the historical data set, since it encompasses a longer time span (1854–2008) and can offer more statistical robustness than the Pathfinder (1985–2008) due to its longer time span. ERSST is chosen over HadISST since it compares better in terms of RMS error and bias to Pathfinder SST during periods of overlap (Figure S1). Only ERSST data after year 1945 were used in the analysis, when the estimated standard error falls to less than 0.4°C at all study locations. While somewhat limiting the statistical advantages of the longer time span, it still confers greater robustness than the Pathfinder (53 years versus 23 years).

### Model ensemble member generation

#### Method 1: individual model ensemble

The bias corrected and scaled SST predictions from each AOGCM that passed the selection criteria were used as input to the coral cover change model. Selection criteria were subjectively defined as models with biases <3°C and seasonal scale differences of <1 standard deviations. This effectively removed 17 of the 41 runs of available model SSTs (across the 20 different AOGCMs) that appeared to be outliers in the multi-model database, at least for SST in the study region. See Table S1 for the model SST selection criteria evaluation statistics. The resulting individual calculations of coral cover change for years 2000–2009 (one for each model passing selection criteria), were then averaged with respect to one another, providing a multi-model mean expressing a ‘most likely’ final outcome among the individual model runs, each considered a possible outcome.

#### Method 2: Monte Carlo simulation ensemble

**Figure 5:**
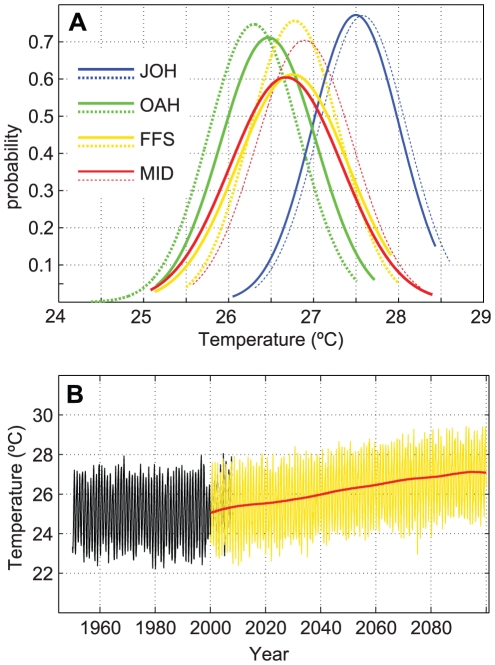
Example of temperature prediction using normal distribution of historic temperatures. Figure 5a represents distributions of August temperatures at study locations from ERSST ver. 3 (solid lines) and Pathfinder SST ver. 5 (dotted lines). Figure 5b: an example of statistical inversion of historic SST (black lines) about the low-pass filtered multi-model mean from all scenario A1B AOGCMs (red line) to produce SST prediction (FFS) (yellow lines).

All AOGCM bias-corrected SST predictions at each location that passed selection criteria (the same as that defined above) were low-pass filtered to remove the model-imposed seasonal fluctuations and then averaged, resulting in a multi-model mean temperature change (increase) for years 2000–2099 for each location. Normal distributions of temperature variance were calculated for each month of the historical time series at each location; different (non-normal) distributions were tested, but did not result in better fit. Future monthly temperature variation about the ensemble mean temperature change is produced using normal random number generation, resulting in a possible future scenario of SST change with seasonal fluctuations constrained by the historical distributions (example, Fig. 5). These SST simulations were used as input to the coral cover change model. The total number of simulations is stopped at 500 at each location; running simulations beyond this number did not result in a significant increase in the variance of possible outcomes of coral cover change. The resulting outcomes express a range of possible outcomes and are averaged to provide a mean expressing a ‘most likely’ net outcome, interpreted similarly as method 1.

### Model Validation and Sensitivity Analysis

To assess the performance of the model, and put the 21^st^ Century predictions of coral cover change into context, model calculations using AOGCMs input for the years 1900–1999 (from Scenario 20C3M) are included in the analysis. Unfortunately, quantitative estimates of coral cover change over time scales of decades do not exist for these years in the region [37]. Comprehensive coral reef monitoring programs were not established until around 1997–2001 [38], [39] in the Hawaiian Archipelago; repeated surveys of individual reef sites in more remote JOH, FFS, and MID did not occur until around 2003–2005 (Kenyon, personal comm.). This makes model optimization and quantitative evaluation difficult, particularly in regard to recovery rates following episodic mortality events.

Bruno and Selig's meta-analysis of existing observational data [37] suggests a coral cover decline of 10–20% in the Hawaiian Archipelago between the 1970s and 1999. This is qualitatively similar to mean decreases in linear extension rates measured from cores and individual corals in a number of studies in the eastern and western Pacific ∼0.89–1.23% year^−1^ for these years [40], [41], [42], although none of them in Hawaii. Lacking better information, the assumption is made that coral cover remained more or less stable at large spatial scales and decadal time scales in the Hawaiian Archipelago for the first 70 or 80 years of the 20^th^ Century, then began a modest (<20%) decline in the last two decades, which is broadly consistent with global-scale findings [37], [43].

To assess the model's sensitivity to various parameters, tests were conducted by varying the most salient model parameters. These parameters are: (1) the (3^rd^-order polynomial) relative growth/temperature relationship (long-term growth and mortality module); (2) the Ω_a_-sensitivity coefficient (long-term CO_2_ effects module); (3) the (2^nd^-order fit) DHM/mortality relationship (episodic heat stress mortality event module); and (4) the starting and ending value of the heat stress (“bleaching”) threshold, e.g. “adaptation” to higher heat stress thresholds (episodic heat stress mortality event module). The relative growth/temperature relationships for each location were varied by randomly perturbing the high and low temperatures of minimum growth by ±0.5°C and the temperature of maximum growth between 0 and 2°C which define the 3^rd^-order polynomial. This effectively randomly changed the skewness, kurtosis and end points of the curves plotted in Fig. 2. The Ω_a_-sensitivity coefficient was varied from 0 to 0.45. The mortality/DHM relationship was varied by using best fits utilizing only the Hawaiian data, only the Caribbean data, and both sets combined (‘MHI+NWHI’, ‘Carib’, and ‘all data’ in Fig. 3). The change in adaptation to higher heat stress thresholds was varied from 0 to 2°C per century.

Each parameter was varied as indicated while the others were held constant during approximately 200 runs of both the individual model ensemble and the Monte Carlo simulation for years 2000–2100. Sensitivities for each parameter were then established by finding the normalized variance (variance of the observations divided by the mean) in the ensemble means and standard deviations of individual ensemble model outcomes associated with that particular parameter. Thus parameters with higher sensitivity in the model would exhibit a higher variance in either the multiple ensemble means or standard deviation of individual ensemble members, or both. To simplify interpretation, these ensemble mean and standard deviation variances were evaluated at (model) years 2050 and 2099, as discussed in the results section and listed in Table S2.

## Results

### Sensitivity analysis

Of the four parameters included for analysis, the models were consistently (at all locations) most sensitive to changing of the heat stress (‘bleaching’) threshold (i.e. ‘adaptation’), particularly in the models' end outcome (i.e. year 2099). Varying this parameter between 0 to 2°C per century (at 0.5°C steps) resulted in an overall (averaged for both the individual model ensemble and the Monte Carlo methods at all locations) ensemble mean normalized variance of 0.05 (5%) for year 2050 and 0.61 (61%) in year 2099. Variance in the standard deviation of individual outcomes (ensemble members) was far smaller (<10% overall). In comparison, model sensitivity to the different DHM/mortality curves (Fig. 3) was much lower (overall ensemble mean variance of 0.4% and 0.6% in 2050 and 2099, respectively) and also lower for the Ω_a_-sensitivity coefficient, particularly in terms of the models' end outcome in year 2100 (overall ensemble mean variance of 3% and 9% in 2050 and 2099, respectively). Unlike when varying the other parameters, perturbing the growth curves (Fig. 2) led to inconsistent behavior. Normalized variance of overall ensemble means was not as low (11% in 2050, 9% in 2099), with a very high variance in the standard deviation of outcomes (59% in 2050, and 49% in 2099). This was due to a small number of highly unstable model ensemble members (leading to coral growth changes of up to 700%) which occurred only at the northern locations (FFS and MID). These unstable ensemble members occurred in less than 10% of the model runs at these locations. If these spurious members are removed, overall normalized model variance is 0.8% in 2050 and 2% in 2099. Data from the sensitivity analysis are summarized in Table S2A.

The sensitivity analysis guided parameter choices used for interpretation of model results. Since variation in outcomes due to the different DHM/mortality curves was very low, all further model results presented here utilize the best 2^nd^ order fit of all DHM/mortality observations (Fig. 3). Also since variation in outcomes due to different growth curves was low in all but a few isolated cases (these are considered in the discussion section) all further results depend on growth curves as defined by SST climatology, without perturbation (Fig. 2). Because of the models' very high dependence on thermal stress threshold and far lower but consistent and linear sensitivity to the Ω_a_ coefficient, two future model parameter scenarios are considered to bound potential outcomes as realistically as possible (within the limitations of the model): (1) no adaptation of thermal stress threshold and a Ω_a_ sensitivity coefficient of 0.3; and (2) a linear increase in heat stress threshold of 1°C and changes in Ω_a_ sensitivity coefficient of 0 (decreasing Ω_a_ will have no effect). These two parameterizations are termed “less resilient” and “more resilient” cases for the remainder of the paper, since (1) assumes corals will have no ability to adapt to increasing water temperatures and will experience a linear decrease in growth rate in response to decreasing Ω_a_ on the order of 30% and (2) corals will adapt their tolerance to heat stress (linearly) by 1°C over the course of the model run (0.1°C/decade) and changes in Ω_a_ will not effect growth rate. The authors considered adaptation of greater than 0.1°C/decade overly optimistic, since any long-term adaptation of corals to temperature stress mortality has yet to be observed [43], [44].

### 20^th^ Century case

**Figure 6:**
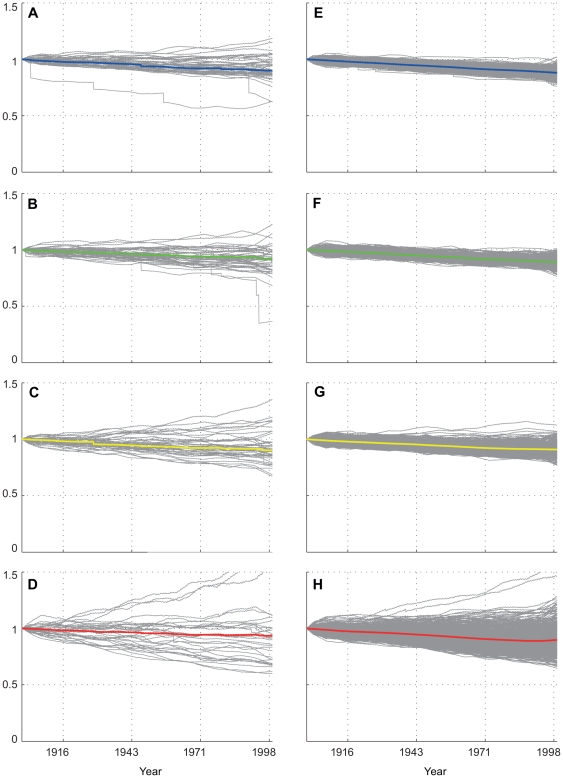
20th century fractional change in coral cover. Individual modal solutions (a–d) plotted for JOH, OAH, FFS, and MID respectively; and Monte Carlo solutions (e–h) for JOH, OAH, FFS, and MID, respectively. Gray lines represent individual solutions from each model (a–d) or PDF solutions (e–h); In this case, corals were assumed to have no temperature adaptation to episodic mortality; Ωa sensitivity at 30% (see methods). Colored lines in each subplot represent ensemble mean.

For this case, the same model parameterization as used for the “less resilient” future case (no ability of corals to adapt to increasing temperature and Ω_a_ sensitivity = 30%) was used. Results from both methods for the 20^th^ century (20C3M) indicate a slow and fairly steady decline in coral cover from 1900 to 2000 at all study locations, with indications of a slightly greater decline in the last two decades (several ensemble members exhibit sudden drops associated with heat-related mortality events towards the end of the century), with a net loss of 5–15% (Fig. 6). The ensemble mean does not indicate an actual trajectory of coral cover change, since year-to-year differences in growth rates and individual episodic bleaching events are averaged out. These means should rather be viewed as a best estimate of long-term (decadal) net change, with the individual solutions representing a range of possible actual trajectories. The (ensemble) mean outcomes for all sites are at least in qualitative agreement with estimates of declines prior to ∼1999 presented for the region [37]. The spread of end-of-20^th^-century outcomes increases with latitude; this is especially apparent for the individual model solutions at MID, where (normal distribution) standard deviation of outcomes is more than double that of OAH and JOH (0.46 versus 0.19 and 0.16, respectively). This is a reflection of both the greater seasonal and intra-annual temperature variability experienced by the northern-most islands in the Hawaiian Archipelago due to their relatively high latitude (28°N) and proximity to the transition zone chlorophyll front (TZCF). This feature marks the surface boundary between the warm surface waters of the North Pacific subtropical gyre and cooler, less stratified waters to the North [45], [46]. Small variations in the position of this boundary in the AOGCMs and the associated larger range of historical temperatures relative to the more southerly study sites are the cause of this greater uncertainty in coral cover change outcomes.

### “Less resilient” future case

**Figure 7:**
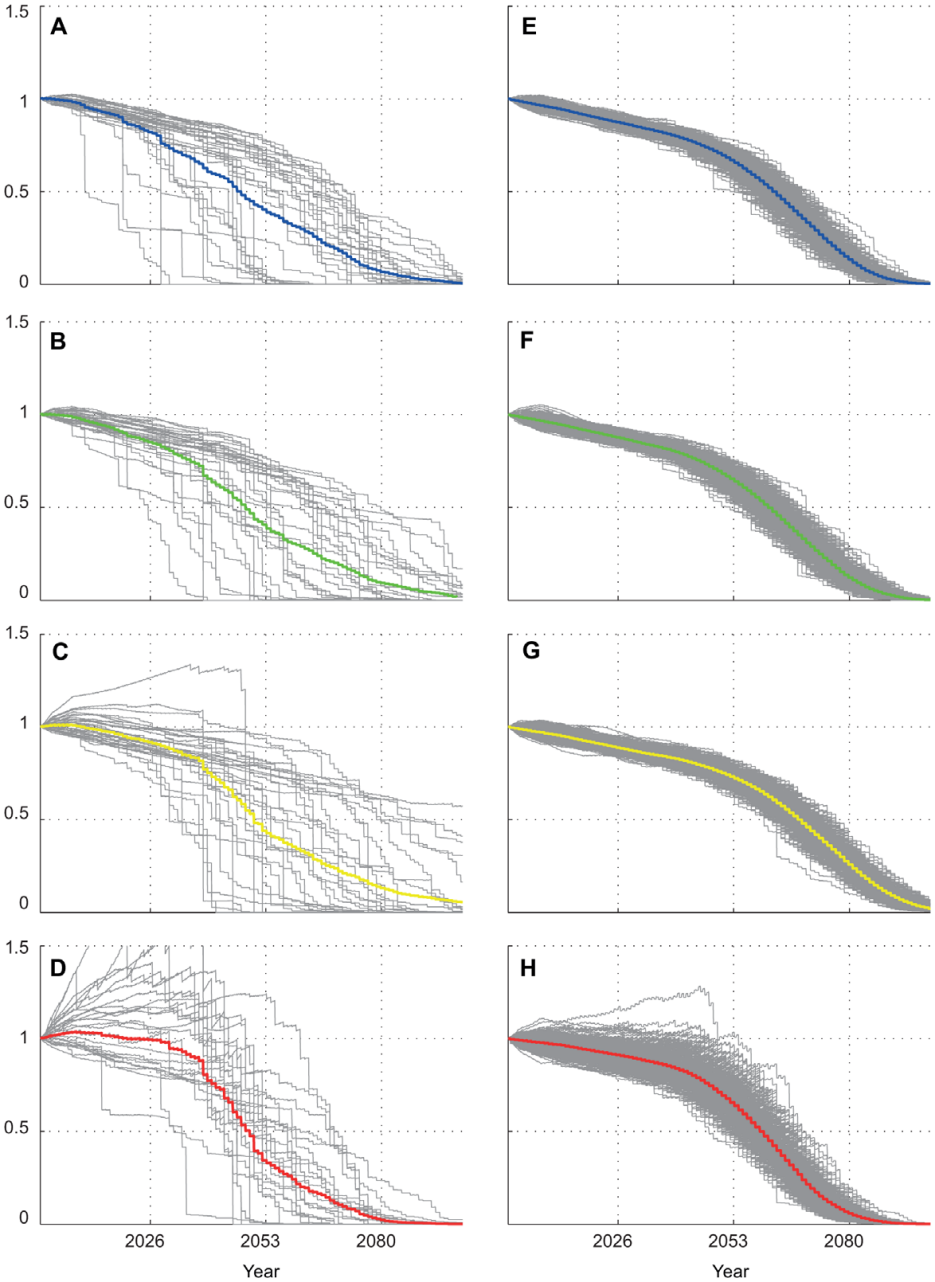
21st century fractional change in coral cover, “less resilient” case. Individual modal solutions (a–d) plotted for JOH, OAH, FFS, and MID respectively; and Monte Carlo solutions (e–h) for JOH, OAH, FFS, and MID, respectively. In this “less resilient” case, corals were assumed to have no temperature adaptation to episodic mortality; Ω_a_ sensitivity at 30%. Colored lines in each subplot represent ensemble mean.

When the same model parameterization as used for the 20^th^ century case (no ability of corals to adapt to increasing temperature and Ω_a_ sensitivity = 30%) were applied to the 21^st^ century A1B scenario, a much different pattern emerges. A rapid ensemble mean decline in coral cover, which becomes precipitous by around 2050, occurred at all sites (Fig. 7). The decline was mainly driven by increasingly frequent and severe heat-stress mortality events, visible as vertical drops in the individual model predictions and the individual Monte Carlo simulations. Probability of a decline in coral cover of >50% by 2050 is very high at JOH, OAH, and MID (probability>0.6), while probabilities of a total loss of viable coral cover (considered a>99% decrease) by 2099 are certain (probability = 1) at JOH and MID and very high at OAH (probability = 0.80) (Table 1). FFS, on the other hand, fares somewhat better, (probability>0.5 that a complete loss of cover will not happen), suggesting that climate predictions of the scenario used in this study (A1B) may confer somewhat greater resilience to the coral reef communities in the vicinity.

**Table 1:** Probabilities of a decrease in coral cover by the years 2050 and 2099 relative to the year 2000.

	“Less resilient” case		“More resilient” case	
	Year 2050: P>50% decrease	Year 2099: P>99% decrease	Year 2050: P>50% decrease	Year 2099: P>50% decrease
JOH	0.67	1.00	0.19	0.57
OAH	0.64	0.80	0.18	0.53
FFS	0.57	0.46	0.18	0.47
MID	0.71	1.00	0.14	0.66

**Figure 8:**
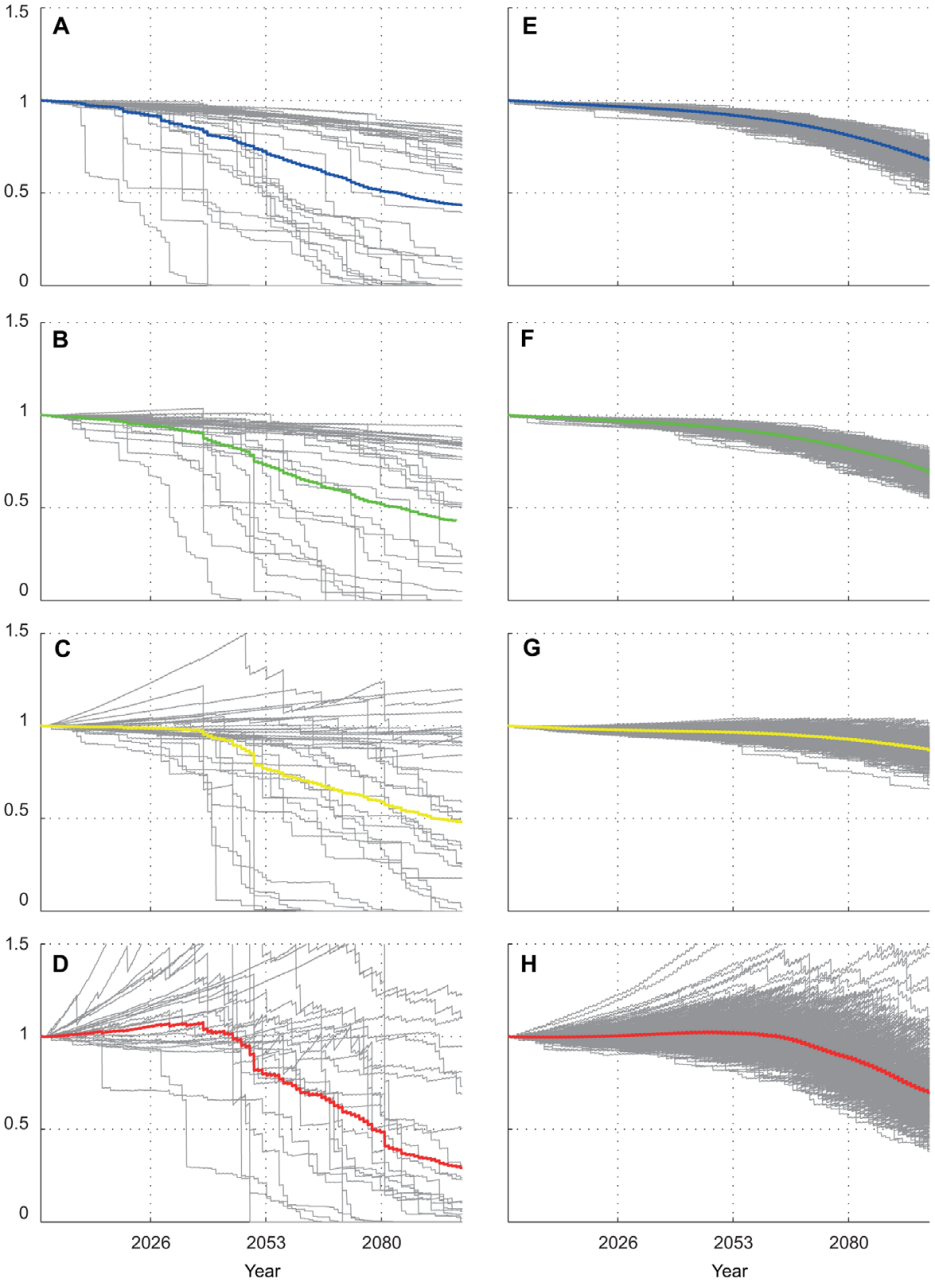
21st century fractional change in coral cover, “more resilient” case. Individual modal solutions (a–d) plotted for JOH, OAH, FFS, and MID respectively; and Monte Carlo solutions (e–h) for JOH, OAH, FFS, and MID, respectively. In this “more resilient” case, the episodic heat stress mortality threshold was allowed to linearly increase 1°C over the century; effects of changing Ω_a_ were ignored (CO2 effects module turned off). Colored lines in each subplot represent ensemble mean.

**Figure 9:**
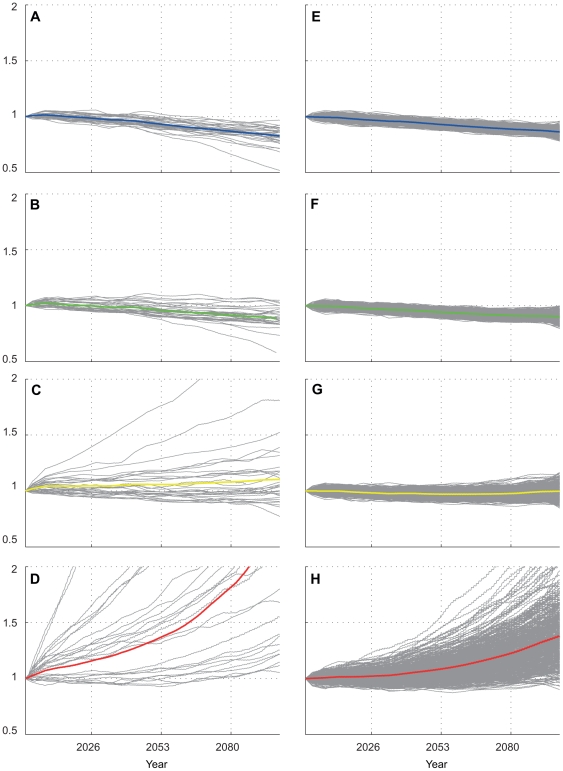
21st century fractional change in coral cover, no episodic mortality. Individual modal solutions (a–d) plotted for JOH, OAH, FFS, and MID respectively; and Monte Carlo solutions (e–h) for JOH, OAH, FFS, and MID, respectively. In this case, the effects of coral bleaching were not accounted for (the episodic heat stress mortality module was turned off). Colored lines in each subplot represent ensemble mean.

Probabilities are calculated using normal distributions of individual model solutions for the respective years. The “less resilient” and “more resilient” cases correspond to Figs. 8a–b and 9a–b, respectively. Note the 2099 “less resilient” case is the probability of a 99% or greater decrease in coral cover, other columns are for 50% or greater decrease.

### “More resilient” future case

If the episodic heat stress mortality threshold is allowed to linearly increase 1°C over the century (unlike the previous two cases, where it was held constant) and the long-term CO_2_ module is turned off, significant 21^st^ century declines still appear to be likely, but far less drastic (Fig. 8). This linear increase in temperature mortality threshold attempts to simulate corals' adaptation to higher levels of heat stress (at 0. 1°C/decade), and assumes that decreasing Ω_a_ will not have a significant effect on calcification rates. Here, coral reef cover predictions do not have such a high likelihood of a 50% decline by mid-century (probability<0.2) and subsequent declines are at much slower rates than the “less resilient” case (Table 1). End of century probabilities of a decrease in coral cover of 50% (relative to 2000 levels) are significantly less likely than the 99% decrease of the “less resilient” case. At FFS and MID, there is actually a small probability of an increase in coral cover, though far less likely than a decrease. Once again FFS fares best here, with lowest likelihoods of decreasing and greatest likelihoods of increasing coral cover.

### Increasing growth rates over time with latitude

The possibility of the northern sites experiencing an increase in coral cover can be explained if the models are run with episodic heat stress mortality module turned off (e.g. no coral bleaching related mortality), as in Fig. 9. Here growth rates increase in the northern areas, as they move under a more favorable area of their respective growth curves (Fig. 2) under warming SST. This effect is particularly apparent at the northern end of the archipelago (MID) where growth rates increase by a factor of 1.5 to 3. The more southerly sites (JOH and OAH) experience more optimal temperatures at the beginning of the 21^st^ century, and thus do not experience a relative increase, rather they decline slightly (Fig. 9). These outcomes are not considered realistic, since they ignore temperature related mortality, but are instructive of changing rates of recovery over time.

### The importance of small scale variability

**Figure 10:**
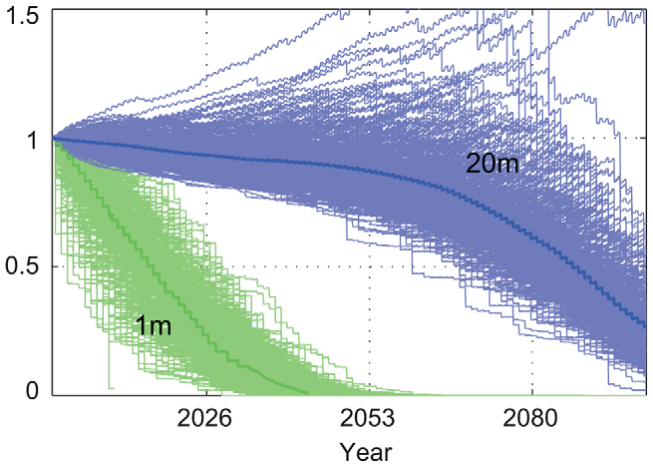
Monte Carlo solutions for fractional change in coral cover using *in situ* temperature measurements. *In situ* temperatures from 1 m and 20 m water depths (as indicated) at Pearl and Hermes Atoll (neighboring MID) were used to constrain and downscale predicted SST (rather than ERSST). In this case, the corals were assumed to have no temperature adaptation to episodic mortality; Ωa sensitivity at 30% (same as the “less resilient” case, Figure 7).

The SST-based predictions presented above do not take into account small-scale (∼<10 km) variations or stratification of water temperatures, nor do they take into account the effects of light attenuation with depth and/or turbidity, which may decrease bleaching severity [10]. The importance of these variations is highlighted by the large difference in coral cover predictions when *in situ* temperature measurements from different depths, one at 1 m and the other at 20 m, at the same reef are used to constrain/downscale model temperatures, rather than historic SST (Fig. 10). Unfortunately these differences only serve to illustrate the importance of small scale variations in temperature at this time, since the length of these *in situ* observations (<6 years) does not characterize seasonal and inter-annual temperature variability in a statistically significant way, and varying light levels are ignored. These differences do provide impetus for the continued maintenance (and expansion) of coral reef observing systems: they may one day provide great insight into small-scale variations at climatological time scales.

## Discussion

The probabilistic approach presented here suggests that, under a regime of warming temperatures over the 21st century (IPCC Scenario A1B), mean growth rates of surviving corals have a high likelihood of increasing significantly (relative to their current values) towards the northernmost end of the Hawaiian Archipelago (e.g. Kure, Midway, Pearl and Hermes Atolls); increasing to a lesser degree towards the center of the chain (e.g. Maro Reef, French Frigate Shoals) and remain roughly stable to the South (the main Hawaiian Islands and Johnston). This increase in relative growth rates from North to South lends qualitative validation to the model's long-term growth and mortality module: as global temperatures warm 2–4°C over the coming century (per A1B), the “Darwin point” [47] would be expected to shift significantly northward, resulting in faster coral calcification rates at higher latitudes. The contribution of increasing growth rates to increasing coral cover will most likely be more than offset by mortality associated with increasing incidence of episodic heat stress events (coral bleaching), especially in the northern end of the archipelago, where projected probabilities of episodic mortality are much higher. Higher incidence and severity of coral bleaching events has already been documented in these northern atolls relative to the rest of the archipelago [26], [27]. If Hawaiian corals are not able to increase their tolerance to future levels of heat stress, model output suggests it is extremely unlikely that viable coral populations will exist in the shallow waters of the Hawaiian Archipelago in 2100. Ensemble averages of individual outcomes suggest precipitous declines in coral cover will likely begin in the northern region sometime between 2030 and 2050, while individual bleaching events are likely to be less severe to the South, leading to more steady decline over the entire century in this region (Fig. 7, Table 1).

However, model outcomes were highly sensitive to increasing the tolerance to future levels of heat stress, e.g. corals will fare much better if they can adapt to episodic mortality either through selection of more thermally tolerant algal symbionts [48], taxonomic succession of more resistant or resilient genera [49], or some combination of these adaptations. This was the single most sensitive parameter in the models. If the threshold for heat stress is allowed to increase at 0.1°C/decade, the model suggests a decline of 25% to 75% (rather than 100%) in coral cover for most locations by the end of the century (Fig. 8, Table 1), possibly less in the northern and center of the chain (due to more rapid recovery). The combination of a relative increase in coral growth rates (compared to the South) and lower risk of mass bleaching (relative to the North) leads to speculation that coral cover in the central archipelago may be more resilient than elsewhere, as evidenced by some of FFS's higher individual coral cover model solutions.

In reality, adaptation to thermal stress, if it occurs on 100-year time scales, will likely not be linear. The “more resilient” adaptive case and the “less resilient” case presented here serve to bound the problem, while the ability of corals to adapt to heat stress remains the subject of debate [43], [44]. This high sensitivity to episodic thermal stress, coupled with sensitivity of growth rates at higher latitudes in some cases, where warming is predicted to be more rapid, points to the need to generally better understand corals' response to changing temperatures.

The inclusion of decreasing Ω_a_ (decreasing ocean pH) does not appear to significantly change the outcomes of the methods presented, beyond lowering long-term estimates of coral cover to some degree (on the order of 20%–30% at the end of century). However, the approach used here is an extreme simplification of complex biogeochemical processes [50], [51]. Therefore it remains poorly understood and poorly modeled, as even archipelago-scale differences in carbon cycles are not accounted for and quantitative impacts on calcification rates remain poorly resolved [2], [50], [52]. For instance, the greater susceptibility of crustose coralline algae calcification rates (a major component of Hawaiian reefs) and reef matrix cementation (relative to corals) may significantly impact coral populations by altering recruitment success, competition for space, and increased bio- and physical erosion of reefs [53], [54]. Despite these shortcomings, the approach used here allows for some estimation of the impacts of decreasing Ω_a_ on calcification rates to be made while details of the interaction of local carbonate processes and coral physiological response remain poorly understood [2], [52].

As stated in the introduction, the temperature and CO_2_ projection used here are large spatial scale projections of (or near) the sea surface only; this ignores small-scale processes that have been shown to lead to very large local differences in bleaching and mortality during observed events (e.g. [10], [29], [55], [56]), and illustrated by Fig. 10. It is therefore reasonably probable, in the context of this study, that even in the “less resilient” case (no ability of corals to adapt to higher temperatures, high Ω_a_ sensitivity), areas of viable coral cover will persist on deeper forereefs or in areas where upwelling of cooler water is occurring.

Due to this modeling effort's simplifying assumptions and a scale that bridges the global and local regimes (as outlined in previous sections), it illustrates the nature and appropriate level of complexity of a regional “building-block” approach to the assessment of future states of global coral reefs. However, it should not be assumed that the predictions of coral cover change presented here are accurate for any particular reef, particularly since import local impacts such as land-based pollution and overfishing are not included, nor is any assumption made that the A1B emissions scenario is particularly valid. The analysis does quantitatively illustrate that (1) current climate modeling science suggests that a large (negative) change in coral cover will occur in 21^st^ century compared to the last, but that (2) there is a significant variability in outcomes, both in space and time, possible even under a single climate change scenario and that this negative change will not necessarily occur everywhere. This variability in outcomes (uncertainty) shows that future attempts to produce quantitative predictions of coral growth and mortality should include a probabilistic approach in which uncertainty is addressed. A logical next step would be to include smaller scale physical and chemical processes and ecosystem dynamics (e.g. integrating predicted succession of different coral taxa [10]), as they become better understood.

## Supporting Information

Figure S1 **Comparison of root mean square differences (RMSE) and mean bias in SST (in °C) between HadlSST v2, ERSST v3, and Pathfinder v5 SST 1985–2007.** Location (JOH, OAH, FFS, MID) given at the top of each row.
(TIF)

Table S1 **Atmosphere-Ocean General Circulation Models (AOGCMs) utilized in this study.** Unless otherwise noted, temperature of surface (TOS) variables were extracted at study locations from the WCRPCMIP3 multi-model dataset for scenarios A1B and 20C3M. “Bias” and “Season. Diff.” columns indicate mean and seasonal differences (in terms of standard deviation) between overlapping periods of 20C3M and ERSST v3 data. Models not passing selection critera (bias>3°C, seasonal standard deviation difference>1) are indicated in the “Notes” column.
(PDF)

Table S2 **Sensitivity Analysis:** (**A**) Sensitivity Summary, and (**B**) all sensitivy runs, ensemble member variance (mu) and ensemble member standard deviations (sigma).
(PDF)
