# Supplementary figures and images for: Projected Changes to Growth and Mortality of Hawaiian Corals over the Next 100 Years
Source: PLoS One. 2011 Mar 29;6(3):e18038. doi: 10.1371/journal.pone.0018038 (PMC3066221; doi:10.1371/journal.pone.0018038)

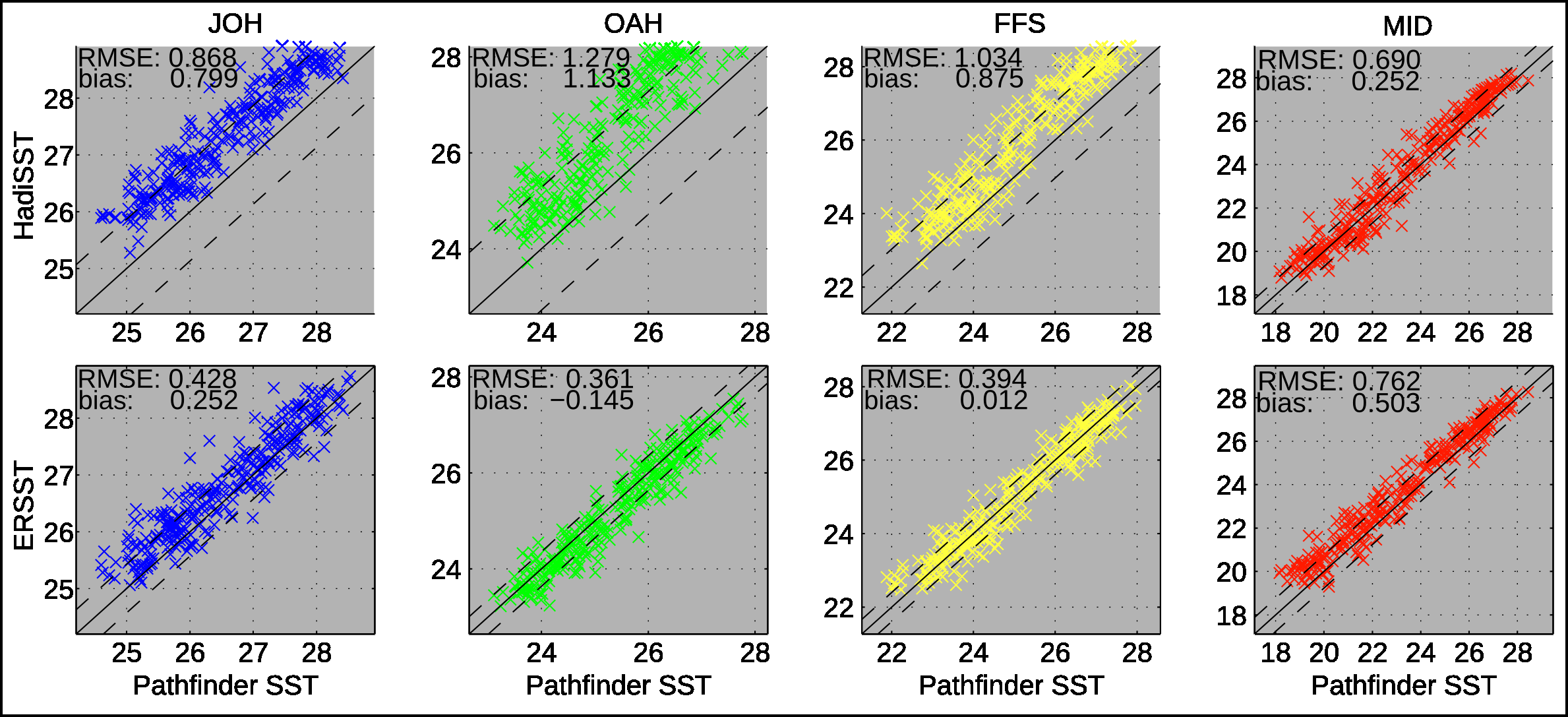

Supplement: Figure S1 — Comparison of root mean square differences (RMSE) and mean bias in SST (in °C) between HadlSST v2, ERSST v3, and Pathfinder v5 SST 1985–2007. Location (JOH, OAH, FFS, MID) given at the top of each row. (TIF) [file pone.0018038.s001.tif]
