# Supplementary material for: Projected Changes to Growth and Mortality of Hawaiian Corals over the Next 100 Years
Source: PLoS One. 2011 Mar 29;6(3):e18038. doi: 10.1371/journal.pone.0018038 (PMC3066221; doi:10.1371/journal.pone.0018038)
Supplement: Table S1 — Atmosphere-Ocean General Circulation Models (AOGCMs) utilized in this study. Unless otherwise noted, temperature of surface (TOS) variables were extracted at study locations from the WCRPCMIP3 multi-model dataset for scenarios A1B and 20C3M. “Bias” and “Season. Diff.” columns indicate mean and seasonal differences (in terms of standard deviation) between overlapping periods of 20C3M and ERSST v3 data. Models not passing selection critera (bias>3°C, seasonal standard deviation difference>1) are indicated in the “Notes” column. (PDF) [file pone.0018038.s002.pdf]

Model Name			Model Spatial Resolution	Location	Grid cell center Lat.	Grid cell center Long.	Bias	Season. diff.	Notes
**BCCR-BCM2.0**	run1	Norway	1.0° x 1.0°	JOH	16.5°	-169.5°	-3.48	0.08	rejected
				OAH	21.5°	-158.5°	-3.43	-0.09	rejected
				FFS	23.5°	-166.5°	-3.28	-0.21	rejected
				MID	28.5°	-177.5°	-2.90	-0.10	
**CCSM3**	run2	USA	0.4° x 1.1°	JOH	16.9°	-169.9°	-0.74	-0.17	
				OAH	21.3°	-157.6°	-1.11	-0.23	
				FFS	23.8°	-166.6°	-0.36	-0.45	
				MID	28.0°	-177.8°	-1.74	-0.34	
**CCSM3**	run5	USA	0.4° x 1.1°	JOH	16.9°	-169.9°	-0.74	-0.17	
				OAH	21.3°	-157.6°	-1.11	-0.23	
				FFS	23.8°	-166.6°	-0.36	-0.45	
				MID	28.0°	-177.8°	-1.74	-0.34	
**CCSM3**	run8	USA	0.4° x 1.1°	JOH	16.9°	-169.9°	-0.74	-0.17	
				OAH	21.3°	-157.6°	-1.11	-0.23	
				FFS	23.8°	-166.6°	-0.36	-0.45	
				MID	28.0°	-177.8°	-1.74	-0.34	
**CNRM-CM3**	run1	France	1.0° x 2.0°	JOH	16.5°	-169.0°	-2.59	0.16	
				OAH	21.5°	-159.0°	-2.95	0.03	
				FFS	23.5°	-167.0°	-2.76	-0.15	
				MID	28.5°	-177.0°	-3.17	-0.12	rejected
**CSIRO-Mk3.0**	run1	Australia	1.9° x 1.9°	JOH	15.9°	-168.8°	-10.02	1.43	rejected
				OAH	21.5°	-158.0°	-7.28	1.13	rejected
				FFS	23.3°	-166.9°	-6.67	0.72	rejected
				MID	28.9°	-178.1°	-3.78	-0.44	rejected
**CSIRO-Mk3.5**	run1	Australia	1.9° x 1.9°	JOH	15.9°	-168.8°	-8.39	1.84	rejected
				OAH	21.5°	-158.0°	-5.95	1.30	rejected
				FFS	23.3°	-166.9°	-4.98	0.94	rejected
				MID	28.9°	-178.1°	-1.68	-0.17	
**ECHAM5/MPI-OM**	run2	Germany	1.0° x 1.0°	JOH	16.5°	-170.0°	-0.30	0.28	
				OAH	21.5°	-158.0°	-0.97	0.07	
				FFS	23.5°	-166.0°	-1.06	-0.02	
				MID	28.5°	-177.0°	-1.70	-0.10	
**ECHO-G**	run1	Germany/ Korea	1.5° x 2.8°	JOH	17.0°	-168.8°	NaN	NaN	unknown extraction
				OAH	NaN	NaN	NaN	NaN	
				FFS	NaN	NaN	NaN	NaN	errors
				MID	NaN	NaN	NaN	NaN	
**ECHO-G**	run2	Germany/ Korea	1.5° x 2.8°	JOH	17.0°	-168.8°	-0.98	-0.08	
				OAH	NaN	NaN	NaN	NaN	unknown extraction
				FFS	NaN	NaN	NaN	NaN	
				MID	NaN	NaN	NaN	NaN	errors
**ECHO-G**	run3	Germany/ Korea	1.5° x 2.8°	JOH	17.0°	-168.8°	-0.94	-0.09	
				OAH	NaN	NaN	NaN	NaN	unknown extraction
				FFS	NaN	NaN	NaN	NaN	
				MID	NaN	NaN	NaN	NaN	errors
**FGOALS-g1.0**	run1	China	1.0° x 1.0°	JOH	17.0°	-170.0°	-0.63	-0.02	
				OAH	21.0°	-158.0°	-0.93	-0.04	
				FFS	24.0°	-166.0°	-1.28	-0.15	
				MID	28.0°	-177.0°	-0.92	-0.32	
**FGOALS-g1.0**	run2	China	1.0° x 1.0°	JOH	17.0°	-170.0°	-0.54	-0.01	
				OAH	21.0°	-158.0°	-0.82	-0.03	
				FFS	24.0°	-166.0°	-1.16	-0.13	
				MID	28.0°	-177.0°	-1.16	-0.31	
**FGOALS-g1.0**	run3	China	1.0° x 1.0°	JOH	17.0°	-170.0°	-0.57	0.00	
				OAH	21.0°	-158.0°	-0.89	-0.02	
				FFS	24.0°	-166.0°	-1.20	-0.13	
				MID	28.0°	-177.0°	-1.10	-0.30	
**GISS-AOM**	run1	USA	3.0° x 4.0°	JOH	16.5°	-170.0°	0.02	-0.33	
				FFS	22.5°	-166.0°	-0.39	-0.22	
				OAH	22.5°	-158.0°	-0.38	0.03	
				MID	28.5°	-178.0°	-1.73	0.08	
**GISS-AOM**	run2	USA	3.0° x 4.0°	JOH	16.5°	-170.0°	-0.01	-0.35	
				FFS	22.5°	-166.0°	-0.37	-0.24	
				OAH	22.5°	-158.0°	-0.38	0.01	
				MID	28.5°	-178.0°	-1.74	0.09	
**GISS-EH**	run1	USA	1.0° x 1.0°	JOH	16.5°	-169.5°	-0.48	0.15	
				OAH	21.5°	-158.5°	-0.49	0.49	
				FFS	23.5°	-166.5°	-0.82	0.39	
				MID	28.5°	-177.5°	-1.64	0.54	
**GISS-EH**	run2	USA	1.0° x 1.0°	JOH	16.5°	-169.5°	-0.49	0.16	
				OAH	21.5°	-158.5°	-0.59	0.50	
				FFS	23.5°	-166.5°	-0.88	0.39	
				MID	28.5°	-177.5°	-1.52	0.53	
**GISS-EH**	run3	USA	1.0° x 1.0°	JOH	16.5°	-169.5°	-0.46	0.17	
				OAH	21.5°	-158.5°	-0.54	0.47	
				FFS	23.5°	-166.5°	-0.81	0.38	
				MID	28.5°	-177.5°	-1.55	0.58	
**GISS-ER**	run1	USA	4.0° x 5.0°	JOH	18.0°	-167.5°	NaN	NaN	unknown extraction
				FFS	22.0°	-167.5°	NaN	NaN	
				OAH	22.0°	-157.5°	NaN	NaN	errors
				MID	30.0°	-177.5°	NaN	NaN	
**GISS-ER**	run2	USA	4.0° x 5.0°	JOH	18.0°	-167.5°	-1.05	-0.21	
				FFS	22.0°	-167.5°	-0.95	-0.54	
				OAH	22.0°	-157.5°	-1.47	-0.24	
				MID	30.0°	-177.5°	-4.62	0.10	rejected

**GISS-ER**	run3	USA	4.0° x 5.0°	JOH	18.0°	-167.5°	-1.11	-0.18	
				FFS	22.0°	-167.5°	-1.05	-0.50	
				OAH	22.0°	-157.5°	-1.60	-0.20	
				MID	30.0°	-177.5°	-4.63	0.09	rejected
**GISS-ER**	run4	USA	4.0° x 5.0°	JOH	18.0°	-167.5°	-1.13	-0.18	
				FFS	22.0°	-167.5°	-1.07	-0.51	
				OAH	22.0°	-157.5°	-1.57	-0.21	
				MID	30.0°	-177.5°	-4.68	0.12	rejected
**GISS-ER**	run5	USA	4.0° x 5.0°	JOH	18.0°	-167.5°	-1.13	-0.18	
				FFS	22.0°	-167.5°	-1.07	-0.53	
				OAH	22.0°	-157.5°	-1.57	-0.21	
				MID	30.0°	-177.5°	-4.65	0.05	rejected
**INGV-SXG**	run1	Italy	1.0° x 1.0°	JOH	16.5°	-169.5°	NaN	NaN	unknown extraction
				OAH	21.5°	-159.0°	NaN	NaN	
				FFS	23.5°	-166.5°	NaN	NaN	errors
				MID	28.5°	-177.5°	NaN	NaN	
**INM-CM3.0**	run1	Russia	2.0° x 2.5°	JOH	16.0°	-170.0°	-1.25	0.60	
				OAH	22.0°	-157.5°	-2.04	0.50	
				FFS	24.0°	-165.0°	-2.27	0.40	
				MID	28.0°	-177.5°	-2.25	0.34	
**IPSL-CM4**	run1	France	1.0° x 2.0°	JOH	16.5°	-169.0°	0.67	0.13	
				OAH	21.5°	-159.0°	0.39	0.06	
				FFS	23.5°	-167.0°	-0.29	0.44	
				MID	28.5°	-177.0°	-4.26	0.58	rejected
**MIROC3.2(hires)**	run1	Japan	0.6° x 1.1°	JOH	16.6°	-169.9°	-1.47	0.03	
				OAH	21.6°	-158.6°	-0.67	-0.07	
				FFS	23.9°	-166.5°	-1.18	-0.14	
				MID	28.4°	-177.8°	-1.70	0.10	
**MIROC3.2(medres)**	run1	Japan	0.9° x 1.4°	JOH	16.4°	-169.5°	-1.89	0.07	
				OAH	21.9°	-158.2°	-2.14	-0.08	
				FFS	23.7°	-166.6°	-1.61	-0.35	
				MID	28.4°	-177.9°	-1.81	-0.44	
**MIROC3.2(medres)**	run2	Japan	0.9° x 1.4°	JOH	16.4°	-169.5°	-1.80	0.04	
				OAH	21.9°	-158.2°	-2.06	-0.09	
				FFS	23.7°	-166.6°	-1.67	-0.35	
				MID	28.4°	-177.9°	-2.08	-0.42	
**MIROC3.2(medres)**	run3	Japan	0.9° x 1.4°	JOH	16.4°	-169.5°	-1.79	0.04	
				OAH	21.9°	-158.2°	-2.02	-0.08	
				FFS	23.7°	-166.6°	-1.53	-0.32	
				MID	28.4°	-177.9°	-1.85	-0.38	
**MRI-CGCM2.3.2**	run1	Japan	1.6° x 2.5°	JOH	16.0°	-170.0°	-0.47	0.14	
				OAH	22.0°	-157.5°	-0.78	0.37	
				FFS	24.0°	-165.0°	-1.18	0.38	
				MID	28.0°	-177.5°	-1.79	0.22	
**MRI-CGCM2.3.2**	run2	Japan	1.6° x 2.5°	JOH	16.0°	-170.0°	-0.47	0.13	
				OAH	22.0°	-157.5°	-0.89	0.38	
				FFS	24.0°	-165.0°	-1.33	0.39	
				MID	28.0°	-177.5°	-1.92	0.22	
**MRI-CGCM2.3.2**	run3	Japan	1.6° x 2.5°	JOH	16.0°	-170.0°	-0.50	0.15	
				OAH	22.0°	-157.5°	-0.89	0.38	
				FFS	24.0°	-165.0°	-1.22	0.37	
				MID	28.0°	-177.5°	-1.71	0.22	
**MRI-CGCM2.3.2**	run4	Japan	1.6° x 2.5°	JOH	16.0°	-170.0°	-0.51	0.13	
				OAH	22.0°	-157.5°	-0.85	0.38	
				FFS	24.0°	-165.0°	-1.15	0.41	
				MID	28.0°	-177.5°	-1.60	0.24	
**MRI-CGCM2.3.2**	run5	Japan	1.6° x 2.5°	JOH	16.0°	-170.0°	-0.45	0.13	
				OAH	22.0°	-157.5°	-0.89	0.40	
				FFS	24.0°	-165.0°	-1.36	0.44	
				MID	28.0°	-177.5°	-1.81	0.25	
**PCM**	run2	USA	1.0° x 1.0°	JOH	16.5°	-169.5°	-0.61	0.82	
				OAH	21.5°	-158.5°	-1.56	0.91	
				FFS	23.5°	-166.5°	-1.38	0.99	
				MID	28.5°	-177.5°	-1.73	1.45	rejected
**PCM**	run3	USA	1.0° x 1.0°	JOH	16.5°	-169.5°	-0.55	0.82	
				OAH	21.5°	-158.5°	-1.56	0.91	
				FFS	23.5°	-166.5°	-1.39	1.04	
				MID	28.5°	-177.5°	-1.80	1.49	rejected
**PCM**	run4	USA	1.0° x 1.0°	JOH	16.5°	-169.5°	-0.47	0.79	
				OAH	21.5°	-158.5°	-1.50	0.90	
				FFS	23.5°	-166.5°	-1.30	0.98	
				MID	28.5°	-177.5°	-1.79	1.45	rejected
**UKMO-HadCM3**	run1	UK	1.3° x 1.3°	JOH	16.9°	-170.0°	-2.42	-0.06	
				OAH	21.9°	-157.5°	-3.01	-0.13	rejected
				FFS	23.1°	-166.3°	-2.92	-0.24	
				MID	28.1°	-177.5°	-2.83	0.34	
**UKMO-HadGEM1**	run1	UK	0.8° x 1.0°	JOH	17.0°	-170.0°	NaN	NaN	unknown extraction
				OAH	21.3°	-158.0°	NaN	NaN	
				FFS	24.1°	-166.0°	NaN	NaN	errors
				MID	28.0°	-177.0°	NaN	NaN	
