# Supplementary material for: Projected Changes to Growth and Mortality of Hawaiian Corals over the Next 100 Years
Source: PLoS One. 2011 Mar 29;6(3):e18038. doi: 10.1371/journal.pone.0018038 (PMC3066221; doi:10.1371/journal.pone.0018038)
Supplement: Table S2 — Sensitivity Analysis: (A) Sensitivity Summary, and (B) all sensitivy runs, ensemble member variance (mu) and ensemble member standard deviations (sigma). (PDF) [file pone.0018038.s003.pdf]

Table S2A

MdT	CaS	LOC'	models 2048 Var mu	models 2048 Var sigma	models 2098 Var mu	models 2098 Var sigma	PDF 2048 Var mu	PDF 2048 Var sigma	PDF 2098 Var mu	PDF 2098 Var sigma	**location mean**	
**Varying MortP:**												
0	0	FFS	0.001	0.001	0.005	0.011	0.002	0.006	0.004	0.003	0.004	
0	0	JOH	0.001	0.001	0.001	0.002	0.004	0.007	0.000	0.000	0.002	
0	0	MID	0.012	0.004	0.000	0.000	0.018	0.004	0.000	0.000	0.005	
0	0	OAH	0.004	0.001	0.036	0.094	0.000	0.007	0.001	0.000	0.018	
1	0	FFS	0.000	0.001	0.002	0.002	0.001	0.002	0.009	0.008	0.003	
1	0	JOH	0.000	0.003	0.001	0.001	0.001	0.005	0.017	0.010	0.005	
1	0	MID	0.004	0.002	0.026	0.030	0.001	0.001	0.099	0.010	0.022	
1	0	OAH	0.000	0.002	0.001	0.002	0.001	0.005	0.019	0.008	0.005	
0	0.3	FFS	0.001	0.001	0.003	0.008	0.002	0.005	0.003	0.002	0.003	
0	0.3	JOH	0.001	0.001	0.000	0.002	0.004	0.005	0.000	0.000	0.002	
0	0.3	MID	0.010	0.003	0.000	0.000	0.016	0.002	0.000	0.000	0.004	
0	0.3	OAH	0.002	0.001	0.001	0.003	0.006	0.005	0.000	0.000	0.002	
**t-mean**			0.003	0.002	0.006	0.013	0.005	0.005	0.013	0.004	0.006	
**Perturbations:**												means excluding FFS&MID outliers:
1	0.000	FFS	0.079	1.617	11.817	78.009	0.001	0.000	0.000161	8.02E-05	11.440	0.000
1	0.000	JOH	0.000	0.000	0.000	0.001	0.000	0.000	7.93E-05	0.000275	0.000	0.000
1	0.000	MID	0.209	0.322	0.283	0.819	0.185	1.106	0.247	0.563	0.467	0.408
1	0.000	OAH	0.000	0.000	0.001	0.001	0.000	0.000	2.74E-05	0.000389	0.000	0.001
0	0.300	FFS	0.010	0.196	0.000	0.001	0.000	0.000	4.35E-07	1.12E-05	0.026	0.000
0	0.300	JOH	0.000	0.000	0.000	0.000	0.000	0.000	5.11E-08	1.61E-07	0.000	0.000
0	0.300	MID	1.425	7.241	0.000	0.000	0.306	0.618	0.000	0.000	2.167	0.000
0	0.300	OAH	0.000	0.000	0.000	0.000	0.000	0.000	3.97E-08	8.99E-08	0.000	0.000
**t-mean**			0.215	1.172	1.513	9.854	0.000	0.000	0.000	0.000	1.763	0.051
**Varying CaS**												
0.5	var	FFS	0.021	0.002	0.103	0.112	0.015	0.007	0.217	0.080	0.070	
0.5	var	JOH	0.020	0.003	0.110	0.165	0.021	0.007	0.054	0.030	0.051	
0.5	var	MID	0.044	0.007	0.010	0.032	0.048	0.003	0.007	0.006	0.020	
0.5	var	OAH	0.022	0.002	0.153	0.217	0.026	0.005	0.051	0.028	0.063	
**t-mean**			0.027	0.003	0.094	0.132	0.027	0.005	0.082	0.036	0.051	
**Varying MdT**												
var	0	FFS	0.039	0.008	0.390	0.122	0.004	0.019	0.675	0.041	0.162	
var	0	JOH	0.045	0.008	0.370	0.215	0.010	0.030	0.654	0.068	0.175	
var	0	MID	0.152	0.009	0.651	0.528	0.049	0.006	1.074	0.191	0.332	
var	0	OAH	0.045	0.013	0.380	0.191	0.016	0.029	0.679	0.067	0.178	
**t-mean**			0.070	0.010	0.448	0.264	0.020	0.021	0.771	0.092	0.212	

Table S2B

Mort	MdT	Ca	CaS	MortP	Pert	LOC'	2048 model mu	2048 model sigma	2098 model mu	2098 model sigma	2048 PDF mu	2048 PDF sigma	2098 PDF mu	2098 PDF sigma
1	0	0	0.00	1	0	FFS	6.791E-01	4.211E-01	7.492E-02	1.948E-01	1.024E+00	4.648E-02	5.391E-03	4.683E-03
1	0	0	0.00	2	0	FFS	6.368E-01	4.334E-01	4.652E-02	1.320E-01	1.025E+00	9.335E-02	3.737E-04	2.818E-04
1	0	0	0.00	3	0	FFS	6.232E-01	4.523E-01	4.687E-02	1.372E-01	1.038E+00	8.558E-02	2.197E-04	2.887E-04
1	0	0	0.00	4	0	FFS	6.140E-01	4.012E-01	3.844E-02	1.018E-01	9.297E-01	9.265E-02	8.696E-04	5.618E-04
1	0	0	0.00	1	0	JOH	5.549E-01	4.285E-01	1.195E-03	3.974E-03	9.438E-01	5.962E-02	7.313E-05	1.823E-04
1	0	0	0.00	2	0	JOH	5.192E-01	4.258E-01	1.924E-04	4.535E-04	8.968E-01	1.212E-01	1.078E-05	1.240E-05
1	0	0	0.00	3	0	JOH	5.133E-01	4.397E-01	7.108E-05	2.522E-04	9.180E-01	1.074E-01	1.170E-06	5.118E-06
1	0	0	0.00	4	0	JOH	4.979E-01	3.909E-01	3.869E-04	8.152E-04	8.018E-01	1.018E-01	5.658E-05	5.478E-05
1	0	0	0.00	1	0	MID	5.596E-01	3.848E-01	2.215E-04	8.829E-04	9.168E-01	1.197E-01	1.729E-05	3.769E-05
1	0	0	0.00	2	0	MID	4.247E-01	3.403E-01	3.154E-05	1.374E-04	7.457E-01	1.644E-01	1.837E-06	2.983E-06
1	0	0	0.00	3	0	MID	4.038E-01	3.591E-01	8.502E-06	3.877E-05	7.718E-01	1.668E-01	9.847E-08	3.412E-07
1	0	0	0.00	4	0	MID	4.194E-01	2.996E-01	6.533E-05	2.300E-04	6.284E-01	1.320E-01	1.369E-05	1.285E-05
1	0	0	0.00	1	0	OAH	6.174E-01	3.935E-01	6.801E-03	1.740E-02	9.234E-01	6.735E-02	1.013E-04	2.102E-04
1	0	0	0.00	2	0	OAH	5.652E-01	4.163E-01	2.210E-03	6.877E-03	8.533E-01	1.227E-01	1.257E-05	1.359E-05
1	0	0	0.00	3	0	OAH	5.580E-01	4.281E-01	1.103E-03	4.111E-03	8.832E-01	1.174E-01	1.475E-06	3.836E-06
1	0	0	0.00	4	0	OAH	5.418E-01	3.754E-01	2.999E-03	8.057E-03	7.475E-01	1.034E-01	6.582E-05	5.510E-05
1	0	1	0.30	1	0	FFS	5.847E-01	3.641E-01	5.317E-02	1.398E-01	8.549E-01	4.123E-02	4.032E-03	3.577E-03
1	0	1	0.30	2	0	FFS	5.479E-01	3.723E-01	3.311E-02	9.528E-02	8.520E-01	8.176E-02	3.039E-04	2.850E-04
1	0	1	0.30	3	0	FFS	5.357E-01	3.885E-01	3.342E-02	9.933E-02	8.643E-01	7.383E-02	1.597E-04	1.939E-04
1	0	1	0.30	4	0	FFS	5.284E-01	3.443E-01	2.723E-02	7.275E-02	7.710E-01	7.955E-02	6.372E-04	3.802E-04
1	0	1	0.30	1	0	JOH	4.709E-01	3.644E-01	8.372E-04	2.780E-03	7.854E-01	5.047E-02	4.531E-05	1.029E-04
1	0	1	0.30	2	0	JOH	4.408E-01	3.628E-01	1.355E-04	3.171E-04	7.465E-01	9.547E-02	7.367E-06	7.781E-06
1	0	1	0.30	3	0	JOH	4.358E-01	3.744E-01	4.975E-05	1.763E-04	7.598E-01	9.266E-02	5.814E-07	1.602E-06
1	0	1	0.30	4	0	JOH	4.230E-01	3.336E-01	2.736E-04	5.719E-04	6.576E-01	8.894E-02	4.042E-05	3.205E-05
1	0	1	0.30	1	0	MID	4.923E-01	3.436E-01	1.601E-04	6.416E-04	7.681E-01	1.063E-01	1.307E-05	3.246E-05
1	0	1	0.30	2	0	MID	3.739E-01	3.065E-01	2.289E-05	1.000E-04	6.133E-01	1.326E-01	9.452E-07	1.139E-06
1	0	1	0.30	3	0	MID	3.540E-01	3.221E-01	6.174E-06	2.821E-05	6.477E-01	1.334E-01	5.840E-08	1.570E-07
1	0	1	0.30	4	0	MID	3.712E-01	2.711E-01	4.728E-05	1.674E-04	5.256E-01	1.142E-01	9.424E-06	8.882E-06
1	0	1	0.30	1	0	OAH	5.260E-01	3.355E-01	4.970E-03	1.285E-02	7.684E-01	5.730E-02	7.280E-05	1.572E-04
1	0	1	0.30	2	0	OAH	4.815E-01	3.541E-01	1.617E-03	5.093E-03	7.139E-01	1.056E-01	8.942E-06	1.166E-05
1	0	1	0.30	3	0	OAH	4.752E-01	3.640E-01	8.120E-04	3.049E-03	7.277E-01	1.010E-01	1.169E-06	4.433E-06
1	0	1	0.30	4	0	OAH	4.618E-01	3.197E-01	2.186E-03	5.951E-03	6.164E-01	9.186E-02	4.440E-05	3.715E-05
1	0	1	0.30	2	1	FFS	9.590E-01	1.423E+00	4.572E-02	1.371E-01	9.581E-01	1.209E-01	4.523E-04	4.362E-04
1	0	1	0.30	2	1	FFS	7.432E-01	5.524E-01	4.610E-02	1.389E-01	9.633E-01	1.260E-01	4.641E-04	3.936E-04
1	0	1	0.30	2	1	FFS	7.320E-01	5.327E-01	4.520E-02	1.305E-01	9.634E-01	1.228E-01	4.492E-04	3.979E-04
1	0	1	0.30	2	1	FFS	8.118E-01	6.882E-01	4.949E-02	1.540E-01	9.609E-01	1.226E-01	4.501E-04	4.352E-04
1	0	1	0.30	2	1	FFS	9.757E-01	1.590E+00	4.258E-02	1.184E-01	9.664E-01	1.230E-01	4.864E-04	6.386E-04
1	0	1	0.30	2	1	FFS	7.836E-01	7.133E-01	4.485E-02	1.266E-01	9.651E-01	1.132E-01	4.647E-04	4.603E-04
1	0	1	0.30	2	1	FFS	7.398E-01	5.555E-01	4.172E-02	1.197E-01	9.640E-01	1.350E-01	4.504E-04	4.361E-04
1	0	1	0.30	2	1	FFS	7.907E-01	6.237E-01	4.416E-02	1.240E-01	9.707E-01	1.215E-01	4.550E-04	4.073E-04
1	0	1	0.30	2	1	FFS	7.780E-01	5.818E-01	4.746E-02	1.355E-01	9.582E-01	1.253E-01	4.802E-04	4.185E-04
1	0	1	0.30	2	1	FFS	7.147E-01	5.301E-01	4.425E-02	1.234E-01	9.710E-01	1.291E-01	4.796E-04	4.490E-04
1	0	1	0.30	2	1	JOH	5.025E-01	4.196E-01	1.675E-04	3.817E-04	7.914E-01	1.028E-01	9.283E-06	1.085E-05
1	0	1	0.30	2	1	JOH	4.965E-01	4.165E-01	1.702E-04	3.950E-04	7.804E-01	1.101E-01	8.827E-06	9.290E-06
1	0	1	0.30	2	1	JOH	4.962E-01	4.131E-01	1.685E-04	3.861E-04	7.851E-01	1.088E-01	9.979E-06	1.285E-05
1	0	1	0.30	2	1	JOH	5.020E-01	4.219E-01	1.727E-04	3.899E-04	7.946E-01	1.022E-01	1.006E-05	1.057E-05
1	0	1	0.30	2	1	JOH	5.064E-01	4.278E-01	1.698E-04	3.887E-04	7.942E-01	1.005E-01	1.034E-05	1.266E-05
1	0	1	0.30	2	1	JOH	4.964E-01	4.169E-01	1.683E-04	3.860E-04	7.851E-01	1.100E-01	1.043E-05	1.214E-05
1	0	1	0.30	2	1	JOH	5.005E-01	4.178E-01	1.690E-04	3.797E-04	7.833E-01	1.107E-01	8.634E-06	1.178E-05
1	0	1	0.30	2	1	JOH	4.961E-01	4.141E-01	1.655E-04	3.756E-04	7.915E-01	1.028E-01	1.057E-05	1.401E-05
1	0	1	0.30	2	1	JOH	4.975E-01	4.203E-01	1.671E-04	3.815E-04	7.869E-01	1.028E-01	1.002E-05	1.120E-05
1	0	1	0.30	2	1	JOH	5.010E-01	4.175E-01	1.732E-04	3.952E-04	7.846E-01	1.039E-01	9.070E-06	1.066E-05
1	0	1	0.30	2	1	MID	6.678E-01	1.429E+00	2.103E-07	7.107E-07	1.332E+00	1.910E+00	2.636E-07	6.661E-07
1	0	1	0.30	2	1	MID	1.723E-01	5.310E-01	1.530E-06	7.392E-06	4.681E-04	5.454E-01	1.157E-06	1.032E-05
1	0	1	0.30	2	1	MID	1.329E-01	4.207E-01	1.244E-08	5.537E-08	2.396E-01	8.229E-01	1.334E-08	1.156E-08
1	0	1	0.30	2	1	MID	6.354E-02	1.528E-01	8.273E-09	3.778E-08	1.347E-02	2.995E-01	6.163E-09	7.541E-08
1	0	1	0.30	2	1	MID	1.536E+00	7.289E+00	9.065E-09	4.431E-08	1.012E-01	4.545E-01	3.240E-06	5.187E-05
1	0	1	0.30	2	1	MID	2.642E-01	8.509E-01	3.832E-05	1.877E-04	3.964E-01	1.241E+00	1.679E-05	1.680E-04
1	0	1	0.30	2	1	MID	3.008E+00	1.328E+01	9.808E-07	4.641E-06	5.906E-01	2.196E-01	4.884E-07	7.686E-07
1	0	1	0.30	2	1	MID	1.303E-01	5.851E-01	8.328E-13	4.080E-12	9.592E-02	1.377E-01	8.426E-13	6.685E-12
1	0	1	0.30	2	1	MID	1.524E-01	4.102E-01	1.594E-07	7.361E-07	3.725E-02	2.433E-01	2.467E-07	3.167E-07
1	0	1	0.30	2	1	MID	1.698E-01	7.739E-01	6.629E-08	3.248E-07	2.516E-01	3.071E-01	9.848E-08	3.284E-07
1	0	1	0.30	2	1	OAH	5.524E-01	4.084E-01	2.297E-03	7.055E-03	7.703E-01	1.219E-01	1.073E-05	1.222E-05
1	0	1	0.30	2	1	OAH	5.466E-01	4.066E-01	2.332E-03	7.494E-03	7.626E-01	1.107E-01	1.133E-05	1.467E-05
1	0	1	0.30	2	1	OAH	5.450E-01	4.042E-01	2.204E-03	6.708E-03	7.674E-01	1.136E-01	1.129E-05	1.409E-05
1	0	1	0.30	2	1	OAH	5.540E-01	4.123E-01	2.405E-03	7.551E-03	7.686E-01	1.116E-01	1.135E-05	1.316E-05
1	0	1	0.30	2	1	OAH	5.538E-01	4.158E-01	2.597E-03	8.696E-03	7.605E-01	1.155E-01	1.296E-05	1.621E-05
1	0	1	0.30	2	1	OAH	5.526E-01	4.041E-01	2.529E-03	7.889E-03	7.657E-01	1.145E-01	1.160E-05	1.403E-05
1	0	1	0.30	2	1	OAH	5.595E-01	4.228E-01	2.385E-03	7.001E-03	7.627E-01	1.158E-01	1.075E-05	1.356E-05
1	0	1	0.30	2	1	OAH	5.527E-01	4.145E-01	2.503E-03	8.290E-03	7.576E-01	1.111E-01	1.119E-05	1.394E-05
1	0	1	0.30	2	1	OAH	5.516E-01	4.117E-01	2.475E-03	7.637E-03	7.657E-01	1.239E-01	1.069E-05	1.269E-05
1	0	1	0.30	2	1	OAH	5.534E-01	4.181E-01	2.275E-03	7.110E-03	7.633E-01	1.165E-01	1.179E-05	1.449E-05
1	0.5	0	0.00	2	0	FFS	8.173E-01	4.274E-01	2.402E-01	4.113E-01	1.134E+00	5.146E-02	2.263E-01	8.744E-02
1	0.5	0	0.00	2	0	JOH	6.843E-01	4.390E-01	1.127E-01	1.714E-01	1.056E+00	6.925E-02	5.391E-02	3.020E-02
1	0.5	0	0.00	2	0	MID	6.985E-01	4.007E-01	1.085E-02	3.399E-02	1.026E+00	1.529E-01	7.154E-03	5.720E-03
1	0.5	0	0.00	2	0	OAH	7.473E-01	4.028E-01	1.677E-01	2.547E-01	1.040E+00	8.138E-02	5.151E-02	2.796E-02
1	0.5	1	0.15	2	0	FFS	5.777E-01	4.189E-01	3.957E-02	1.167E-01	9.482E-01	8.101E-02	1.935E-04	2.494E-04
1	0.5	1	0.15	2	0	JOH	4.729E-01	4.057E-01	5.947E-05	2.108E-04	8.368E-01	9.548E-02	7.207E-07	1.965E-06
1	0.5	1	0.15	2	0	MID	3.779E-01	3.396E-01	7.245E-06	3.307E-05	6.987E-01	1.482E-01	8.510E-08	2.850E-07
1	0.5	1	0.15	2	0	OAH	5.149E-01	3.946E-01	9.461E-04	3.541E-03	7.991E-01	1.098E-01	9.192E-07	2.010E-06
1	0.5	1	0.30	2	0	FFS	5.694E-01	3.714E-01	3.235E-02	8.603E-02	8.456E-01	8.385E-02	7.260E-04	4.486E-04
1	0.5	1	0.30	2	0	JOH	4.589E-01	3.610E-01	3.253E-04	6.827E-04	7.286E-01	9.766E-02	4.617E-05	3.372E-05
1	0.5	1	0.30	2	0	MID	3.944E-01	2.845E-01	5.558E-05	1.962E-04	5.783E-01	1.184E-01	1.097E-05	1.008E-05
1	0.5	1	0.30	2	0	OAH	5.002E-01	3.463E-01	2.560E-03	6.924E-03	6.887E-01	9.651E-02	5.069E-05	3.954E-05
1	0.5	1	0.45	2	0	FFS	6.300E-01	3.912E-01	6.311E-02	1.650E-01	9.348E-01	4.075E-02	4.667E-03	4.184E-03
1	0.5	1	0.45	2	0	JOH	5.112E-01	3.950E-01	1.000E-03	3.324E-03	8.603E-01	4.959E-02	6.580E-05	1.278E-04
1	0.5	1	0.45	2	0	MID	5.246E-01	3.629E-01	1.883E-04	7.527E-04	8.483E-01	1.181E-01	1.859E-05	6.243E-05
1	0.5	1	0.45	2	0	OAH	5.698E-01	3.632E-01	5.814E-03	1.495E-02	8.484E-01	6.026E-02	9.498E-05	2.069E-04
1	1	0	0.00	1	0	FFS	9.432E-01	3.556E-01	6.430E-01	5.268E-01	1.093E+00	2.236E-02	1.112E+00	6.829E-02
1	1	0	0.00	2	0	FFS	9.622E-01	3.749E-01	5.904E-01	5.907E-01	1.158E+00	3.050E-02	1.159E+00	1.314E-01

1	1	0	0.00	3	0	FFS	9.489E-01	4.038E-01	5.906E-01	5.934E-01	1.162E+00	2.940E-02	1.187E+00	1.224E-01
1	1	0	0.00	4	0	FFS	9.432E-01	3.631E-01	5.570E-01	5.645E-01	1.141E+00	3.953E-02	9.623E-01	1.309E-01
1	1	0	0.00	1	0	JOH	8.388E-01	3.211E-01	5.435E-01	4.535E-01	1.050E+00	1.580E-02	8.591E-01	7.077E-02
1	1	0	0.00	2	0	JOH	8.318E-01	3.803E-01	5.444E-01	4.887E-01	1.106E+00	4.044E-02	7.777E-01	1.453E-01
1	1	0	0.00	3	0	JOH	8.299E-01	3.880E-01	5.478E-01	4.940E-01	1.110E+00	3.321E-02	8.121E-01	1.395E-01
1	1	0	0.00	4	0	JOH	8.095E-01	3.725E-01	4.988E-01	4.519E-01	1.081E+00	4.738E-02	5.973E-01	1.170E-01
1	1	0	0.00	1	0	MID	1.082E+00	4.689E-01	3.786E-01	5.091E-01	1.148E+00	9.548E-02	8.466E-01	2.090E-01
1	1	0	0.00	2	0	MID	9.895E-01	4.347E-01	2.373E-01	3.536E-01	1.184E+00	1.213E-01	4.572E-01	1.701E-01
1	1	0	0.00	3	0	MID	9.714E-01	4.735E-01	2.424E-01	3.809E-01	1.188E+00	1.157E-01	4.878E-01	1.871E-01
1	1	0	0.00	4	0	MID	9.431E-01	4.033E-01	1.869E-01	2.524E-01	1.108E+00	1.216E-01	3.094E-01	1.133E-01
1	1	0	0.00	1	0	OAH	8.991E-01	2.885E-01	5.824E-01	4.680E-01	1.053E+00	2.180E-02	8.753E-01	7.643E-02
1	1	0	0.00	2	0	OAH	8.941E-01	3.412E-01	5.782E-01	5.251E-01	1.111E+00	4.407E-02	7.856E-01	1.431E-01
1	1	0	0.00	3	0	OAH	8.938E-01	3.524E-01	5.785E-01	5.312E-01	1.111E+00	3.784E-02	8.073E-01	1.422E-01
1	1	0	0.00	4	0	OAH	8.728E-01	3.302E-01	5.357E-01	4.871E-01	1.074E+00	5.365E-02	5.957E-01	1.202E-01
1	1	0	0.00	2	1	FFS	1.305E+00	6.475E-01	1.382E+00	1.781E+00	1.313E+00	1.243E-01	1.915E+00	6.463E-01
1	1	0	0.00	2	1	FFS	2.116E+00	4.812E+00	1.837E+01	9.368E+01	1.309E+00	1.064E-01	1.876E+00	5.334E-01
1	1	0	0.00	2	1	FFS	1.334E+00	8.095E-01	1.500E+00	2.033E+00	1.302E+00	1.055E-01	1.837E+00	5.036E-01
1	1	0	0.00	2	1	FFS	1.794E+00	2.928E+00	7.198E+00	3.072E+01	1.398E+00	2.012E+00	3.145E+01	6.604E+02
1	1	0	0.00	2	1	FFS	1.729E+00	2.424E+00	4.964E+00	2.009E+01	1.308E+00	1.178E-01	1.901E+00	6.208E-01
1	1	0	0.00	2	1	FFS	1.569E+00	1.627E+00	3.054E+00	7.941E+00	1.306E+00	1.135E-01	1.880E+00	5.270E-01
1	1	0	0.00	2	1	FFS	4.144E+00	1.540E+01	2.293E+02	1.247E+03	1.296E+00	1.125E-01	1.830E+00	7.278E-01
1	1	0	0.00	2	1	FFS	1.455E+00	9.028E-01	2.110E+00	3.386E+00	1.308E+00	1.136E-01	1.871E+00	6.437E-01
1	1	0	0.00	2	1	FFS	2.327E+00	5.967E+00	2.829E+01	1.478E+02	1.310E+00	1.199E-01	1.887E+00	6.148E-01
1	1	0	0.00	2	1	FFS	1.356E+00	7.337E-01	1.363E+00	1.691E+00	1.309E+00	1.240E-01	1.892E+00	6.570E-01
1	1	0	0.00	2	1	JOH	9.478E-01	4.396E-01	8.009E-01	7.443E-01	1.176E+00	4.753E-02	1.017E+00	2.054E-01
1	1	0	0.00	2	1	JOH	9.495E-01	4.555E-01	8.148E-01	7.846E-01	1.173E+00	4.542E-02	1.022E+00	1.840E-01
1	1	0	0.00	2	1	JOH	9.500E-01	4.456E-01	8.060E-01	7.400E-01	1.175E+00	4.782E-02	1.013E+00	2.009E-01
1	1	0	0.00	2	1	JOH	9.397E-01	4.411E-01	7.912E-01	7.667E-01	1.177E+00	4.664E-02	1.023E+00	1.978E-01
1	1	0	0.00	2	1	JOH	9.374E-01	4.369E-01	7.754E-01	7.104E-01	1.176E+00	4.963E-02	1.021E+00	1.902E-01
1	1	0	0.00	2	1	JOH	9.426E-01	4.449E-01	7.944E-01	7.507E-01	1.174E+00	4.981E-02	1.029E+00	1.876E-01
1	1	0	0.00	2	1	JOH	9.365E-01	4.396E-01	7.902E-01	7.492E-01	1.172E+00	5.152E-02	9.991E-01	1.949E-01
1	1	0	0.00	2	1	JOH	9.410E-01	4.403E-01	7.839E-01	7.110E-01	1.174E+00	4.818E-02	1.025E+00	1.934E-01
1	1	0	0.00	2	1	JOH	9.358E-01	4.320E-01	7.747E-01	7.075E-01	1.175E+00	4.690E-02	1.020E+00	2.028E-01
1	1	0	0.00	2	1	JOH	9.410E-01	4.407E-01	7.895E-01	7.251E-01	1.174E+00	4.828E-02	1.007E+00	2.042E-01
1	1	0	0.00	2	1	MID	4.372E-01	9.457E-01	4.147E-01	1.228E+00	6.071E-01	1.152E+00	1.478E-02	1.021E+00
1	1	0	0.00	2	1	MID	2.044E-01	5.581E-01	3.484E-01	1.676E+00	2.473E-02	4.968E-01	2.169E-01	1.695E-01
1	1	0	0.00	2	1	MID	7.508E-02	3.678E-01	0.000E+00	0.000E+00	1.001E-01	2.327E-01	0.000E+00	0.000E+00
1	1	0	0.00	2	1	MID	9.167E-01	1.902E+00	8.331E-01	2.458E+00	5.641E-02	2.123E+00	1.590E+00	7.220E-01
1	1	0	0.00	2	1	MID	3.132E-01	7.634E-01	4.170E-03	1.460E-02	5.010E-01	8.514E-01	4.408E-04	5.382E-03
1	1	0	0.00	2	1	MID	6.580E-01	1.180E+00	2.687E-01	9.568E-01	2.497E-01	1.325E+00	2.753E-01	4.439E-01
1	1	0	0.00	2	1	MID	2.525E-01	6.999E-01	2.224E-01	8.451E-01	1.530E-01	1.208E+00	2.892E-01	4.088E-01
1	1	0	0.00	2	1	MID	7.898E-02	3.869E-01	0.000E+00	0.000E+00	8.159E-03	7.688E-01	0.000E+00	0.000E+00
1	1	0	0.00	2	1	MID	4.229E-01	1.814E+00	2.572E-02	1.214E-01	4.690E-03	1.761E+00	5.900E-03	3.529E-02
1	1	0	0.00	2	1	MID	1.355E-01	6.638E-01	2.967E-01	1.453E+00	1.413E-01	1.144E+00	7.390E-02	2.826E+00
1	1	0	0.00	2	1	OAH	1.025E+00	3.982E-01	8.524E-01	7.887E-01	1.194E+00	5.956E-02	1.051E+00	2.193E-01
1	1	0	0.00	2	1	OAH	1.017E+00	4.054E-01	8.283E-01	7.494E-01	1.190E+00	6.022E-02	1.058E+00	2.208E-01
1	1	0	0.00	2	1	OAH	1.014E+00	3.993E-01	8.395E-01	7.679E-01	1.187E+00	6.049E-02	1.058E+00	2.224E-01
1	1	0	0.00	2	1	OAH	1.027E+00	3.967E-01	8.404E-01	7.624E-01	1.192E+00	6.323E-02	1.052E+00	2.226E-01
1	1	0	0.00	2	1	OAH	1.018E+00	3.983E-01	8.389E-01	7.503E-01	1.193E+00	5.889E-02	1.061E+00	2.157E-01
1	1	0	0.00	2	1	OAH	1.029E+00	4.106E-01	8.636E-01	8.199E-01	1.193E+00	5.676E-02	1.049E+00	2.024E-01
1	1	0	0.00	2	1	OAH	1.023E+00	3.956E-01	8.519E-01	8.015E-01	1.189E+00	6.131E-02	1.050E+00	2.041E-01
1	1	0	0.00	2	1	OAH	1.004E+00	3.929E-01	8.149E-01	7.496E-01	1.195E+00	5.874E-02	1.050E+00	2.332E-01
1	1	0	0.00	2	1	OAH	1.039E+00	4.208E-01	9.039E-01	8.448E-01	1.191E+00	6.215E-02	1.061E+00	2.226E-01
1	1	0	0.00	2	1	OAH	1.046E+00	4.269E-01	8.986E-01	8.125E-01	1.191E+00	6.186E-02	1.063E+00	2.230E-01
1	1.5	0	0.00	2	0	FFS	1.062E+00	3.113E-01	1.028E+00	6.508E-01	1.168E+00	2.547E-02	1.396E+00	6.793E-02
1	1.5	0	0.00	2	0	JOH	9.409E-01	3.141E-01	7.758E-01	5.217E-01	1.124E+00	2.132E-02	1.179E+00	5.514E-02
1	1.5	0	0.00	2	0	MID	1.252E+00	4.829E-01	9.314E-01	8.844E-01	1.258E+00	1.017E-01	1.570E+00	3.237E-01
1	1.5	0	0.00	2	0	OAH	1.005E+00	2.660E-01	8.650E-01	5.314E-01	1.133E+00	2.118E-02	1.218E+00	5.938E-02
